# Effectiveness and safety of dolutegravir and raltegravir for treating children and adolescents living with HIV: a systematic review

**DOI:** 10.1002/jia2.25970

**Published:** 2022-11-14

**Authors:** Claire L. Townsend, John O'Rourke, Edith Milanzi, Intira Jeannie Collins, Ali Judd, Hannah Castro, Marissa Vicari, Julie Jesson, Valériane Leroy, Françoise Renaud, Martina Penazzato

**Affiliations:** ^1^ Consultants to the World Health Organization Geneva Switzerland; ^2^ MRC Clinical Trials Unit at UCL University College London London UK; ^3^ International AIDS Society Geneva Switzerland; ^4^ Center for Epidemiology and Research in POPulation Health (CERPOP), Inserm, Université de Toulouse Université Paul Sabatier Toulouse France; ^5^ HIV Department World Health Organization Geneva Switzerland

**Keywords:** children, drug‐related side effects and adverse reactions, integrase inhibitors, HIV, systematic review, treatment outcome

## Abstract

**Introduction:**

Globally about 1.7 million children were living with HIV in 2020. Two integrase strand transfer inhibitors, dolutegravir and raltegravir, are increasingly used in children. We conducted a systematic review to assess the effectiveness and safety of dolutegravir and raltegravir in children and adolescents living with HIV, aged 0–19 years.

**Methods:**

Sources included MEDLINE, Embase, the Cochrane Library, clinical trial registries, abstracts from key conferences and reference list searching. Observational studies and clinical trials published January 2009–March 2021 were eligible. Outcomes included efficacy/effectiveness (CD4 counts and viral load) and/or safety outcomes (mortality, grade 3/4 adverse events and treatment discontinuation) through 6 months or more post‐treatment initiation. Risk of bias was assessed using previously published tools appropriate for the study design. Narrative syntheses were conducted.

**Results and discussion:**

In total, 3626 abstracts and 371 papers were screened. Eleven studies, including 2330 children/adolescents, reported data on dolutegravir: one randomized controlled trial (RCT; low risk of bias), one single‐arm trial (unclear risk of bias) and nine cohort studies (three low risk of bias, two unclear risk and four high risk). Ten studies, including 649 children/adolescents receiving raltegravir, were identified: one RCT (low risk of bias), one single‐arm trial (low risk of bias) and eight cohort studies (four low risk of bias, three unclear risk and one high risk). Viral suppression levels in children/adolescents at 12 months were high (>70%) in most studies assessing dolutegravir (mostly second‐ or subsequent‐line, or mixed treatment lines), and varied from 42% (5/12) to 83% (44/53) at 12 months in studies assessing raltegravir (mostly second‐ or subsequent‐line). Across all studies assessing dolutegravir or raltegravir, grade 3/4 adverse events (clinical and/or laboratory) were reported in 0–50% of subjects, few resulted in discontinuation, few were drug related and no deaths were attributed to either drug.

**Conclusions:**

These reassuring findings suggest that dolutegravir and raltegravir are effective and safe as preferred regimens in children and adolescents living with HIV. With the rollout of dolutegravir in paediatric populations already underway, it is critical that data are collected on safety and effectiveness in infants, children and adolescents, including on longer‐term outcomes, such as weight and metabolic changes.

## 1 INTRODUCTION

In 2020, approximately 1.7 million children aged 0–14 years were living with HIV worldwide [1]. The World Health Organization (WHO) recommends that all children and adolescents living with HIV start antiretroviral therapy (ART) regardless of CD4 cell count or disease stage [2]. Since treatment is life‐long, optimizing therapeutic regimens to maximize effectiveness and tolerability, and minimize side effects is critical to achieving the durability of treatment and favourable health outcomes in children and adolescents.

Dolutegravir is an integrase strand transfer inhibitor (integrase inhibitor) that has a high genetic barrier to developing HIV drug resistance, and few drug–drug interactions compared with other antiretrovirals [3, 4]. Dolutegravir is a key component of first‐line and second‐line treatment for adults living with HIV and is also recommended as the preferred option for all children aged ≥4 weeks and weighing ≥3 kg [5]. Another integrase inhibitor, raltegravir, is approved for use from birth in full‐term neonates weighing ≥2 kg and is recommended as the preferred regimen for neonates, and the alternative integrase inhibitor regimen for infants aged ≥4 weeks [5, 6]. Two other integrase inhibitors, elvitegravir and bictegravir, are approved for paediatric use in fixed‐dose formulations, but these are not currently recommended by WHO.

Despite the documented benefits of integrase inhibitors, sleep disorders have been reported in adults on dolutegravir, and there are questions around weight gain in adults on dolutegravir or raltegravir [7, 8, 9]. With the widespread adoption of integrase inhibitor‐based regimens for first‐line and subsequent lines of treatment, monitoring short‐ and long‐term health outcomes is critical. As new paediatric formulations of dolutegravir become available, it is particularly important to establish ongoing effectiveness and safety in real‐world clinical settings in younger populations. To date, no systematic reviews have summarized the use of integrase inhibitors in children and adolescents. We conducted this systematic review on the effectiveness and safety of dolutegravir and raltegravir in neonates, infants, children and adolescents to support the development of the 2021 WHO consolidated guidelines on HIV prevention, testing, treatment, service delivery and monitoring [5], and to inform the prioritization of future optimal drug formulations for children and adolescents [10].

## 2 METHODS

This systematic review followed Centre for Reviews and Dissemination guidance [11], and is reported according to Preferred Reporting Items for Systematic Reviews and Meta‐Analyses (PRISMA) guidelines [12] (PROSPERO number CRD42020204432) [13]. The review was developed to assess five drugs: dolutegravir, raltegravir, darunavir, lopinavir (solid formulations) and tenofovir alafenamide. This paper presents the results pertaining to dolutegravir and raltegravir.

### 2.1 Data sources and search strategies

Searches were run in MEDLINE, MEDLINE In‐Process, MEDLINE E‐pub ahead of print (via Ovid), Embase (via Ovid) and The Cochrane Library using both free‐text terms and index terms (File S1). Grey literature searches involved hand‐screening of reference lists from all included studies and HIV treatment guidelines, and searches of clinical trial registries. Additionally, databases of abstracts from selected conferences occurring January 2018–March 2021 were searched (File S1).

English‐ or French‐language publications were eligible if they reported on children and/or adolescents living with HIV aged 0–19 years, receiving dolutegravir or raltegravir for first‐line or subsequent‐line of therapy (Table 1). Studies were only included if they presented results stratified by drug, or if at least 80% of the treatment group were on one of the two drugs, and if they reported efficacy or effectiveness and/or safety outcomes through 6 months post‐treatment initiation. Where eligibility of a study was unclear, authors were contacted for more information. Observational studies and clinical trials published between 1 January 2009 (before first approval date for use of the drugs in children and adolescents) and 21 March 2021 were included. Case studies, letters and reviews were excluded. The following studies were also excluded: those reporting outcomes in infants exposed to HIV *in utero* or through breastfeeding (where the drugs were used for prophylaxis rather than treatment); those reporting only pharmacokinetic parameters or drug–drug interactions; and those reporting only on adult populations aged ≥18 years and/or pregnant women.

**Table 1:** Study eligibility criteria and data extracted

PICO criteria	Description
Population	Children and adolescents (aged 0–19^a^ years) living with HIV who were on first‐line or subsequent line of treatment
Intervention	Dolutegravir or raltegravir in combination with any other antiretroviral therapies recommended for use in a paediatric or adolescent population
Comparator	Dolutegravir, raltegravir, darunavir, lopinavir, or tenofovir alafenamide, or no comparator (i.e. single‐arm trials were included)^b^
Outcomes	*Primary outcomes* Efficacy/effectiveness measured through 6 months follow‐up or more CD4 cell counts or percent HIV‐1 RNA viral load Safety measured through 6 months follow‐up or more Mortality Treatment discontinuation or change and reasons Grade 3/4 adverse events and their association with antiretroviral drugs, including hospitalization *Secondary outcomes* Safety measured through 6 months follow‐up or more Hospitalization Growth and weight gain Diabetes Bone health Renal function Hypersensitivity reactions Lipid levels (cholesterol and triglycerides; change from baseline)
Study design	Observational studies or clinical trials (case studies were excluded)
Publication period	Published between 1 January 2009 and date of searches^c^
Language	English and French language publications

Abbreviation: PICO, population, intervention, comparator, outcome.

^a^ Studies reporting on an adult population that included subjects aged ≥18 years only were excluded, as were studies assessing the effect of exposure to antiretroviral drugs in pregnancy or postnatally.

^b^ For randomized controlled trials only, results for comparator arms were extracted regardless of the comparator treatment.

^c^ Searches were initially run in August 2020 and then updated in March 2021.

### 2.2 Article screening and data extraction

All articles (abstracts and papers) were screened in duplicate against the eligibility criteria by three reviewers (JOR, CT and EM), and results were recorded by each reviewer independently. Disagreements were resolved by discussion and with other members of the project team where necessary. Data from selected articles were extracted into a standard Microsoft Excel form by a single reviewer. Extracted data were independently checked by a second reviewer. Likewise, risk of bias was assessed and recorded by one reviewer and checked by a second reviewer, using the Cochrane Risk of Bias Tool (Version 2) for randomized controlled trials (RCTs), the National Institutes of Health quality assessment tool for non‐randomized interventional studies and the CLARITY tool for cohort studies. The list of questions for each tool is presented in Table 2.

**Table 2:** List of domains or questions used to assess risk of bias for randomized controlled trials, single‐arm trials and observational studies

Randomized controlled trials^a^
Domain 1: Risk of bias from the randomization process
Domain 2: Risk of bias due to deviations from the intended interventions
Domain 3: Missing outcome data
Domain 4: Risk of bias in measurement of the outcome
Domain 5: Risk of bias in selection of the reported result
Single‐arm trials^b^
Qu 1. Was the study question or objective clearly stated?
Qu 2. Were eligibility/selection criteria for the study population prespecified and clearly described?
Qu 3. Were the participants in the study representative of those who would be eligible for the intervention in the general or clinical population of interest?
Qu 4. Were all eligible participants who met the prespecified entry criteria enrolled?
Qu 5. Was the sample size sufficiently large to provide confidence in the findings?
Qu 6. Was the intervention clearly described and delivered consistently across the study population?
Qu 7. Were the outcome measures prespecified, clearly defined, valid, reliable and assessed consistently across all study participants?
Qu 8. Were the people assessing the outcomes blinded to the participants' interventions?
Qu 9. Was the loss to follow‐up after baseline 20% or less? Were those lost to follow‐up accounted for in the analysis?
Qu 10. Did the statistical methods examine changes in outcome measures from before to after the intervention? Were statistical tests done that provided *p* values for the pre‐to‐post changes?
Qu 11. Were outcome measures of interest taken at baseline and multiple times after the intervention?
Qu 12. If the intervention was conducted at a group level, did the statistical analysis take into account the use of individual‐level data to determine effects at the group level?
Observational studies^c^
Qu 1. Was the study population selected in an appropriate way?
Qu 2. Can we be confident in the assessment of exposure?
Qu 3. Can we be confident that the outcome of interest was not present at start of study for (a) effectiveness and (b) safety?
Qu 4. For comparative studies: did the study match exposed and unexposed for all variables that are associated with the outcome of interest or did the statistical analysis adjust for these prognostic variables?
Qu 5. Can we be confident in the assessment of the presence or absence of prognostic factors?
Qu 6. Can we be confident in the assessment of outcome for (a) effectiveness and (b) safety?
Qu 7. Was the follow up of cohorts adequate? (both duration and completeness of follow‐up, i.e. are all subjects accounted for?)
Qu 8. For comparative studies: were co‐interventions similar between groups?

Questions taken from:

^a^ the Cochrane Risk of Bias Tool (Version 2) for randomized controlled trials;

^b^ the National Institutes of Health quality assessment tool for non‐randomized interventional studies; and

^c^ the CLARITY tool for cohort studies.

Data on study characteristics, subject characteristics, efficacy/effectiveness and safety were extracted (Table 1). The primary outcomes were CD4 counts, HIV RNA viral load, mortality, grade 3/4 adverse events and treatment discontinuation. Additional outcomes are listed in File S1. For binary and categorical outcomes, number and proportion of subjects with the event were extracted, or estimated using available data, while for continuous outcomes, mean, median, range, interquartile range (IQR), change from baseline (start of study drug), regression coefficients and 95% confidence intervals (CI) were extracted. Data reported in figures only were extracted using the online tool “Web Plot Digitizer” [14].

### 2.3 Definitions

Infants were defined as <12 months old, children as 1 to under 12 years old and adolescents as 12–19 years old (in line with age categories used in identified studies). Subjects were defined as being on second‐ or subsequent‐line of ART if they had previously received regimens containing drugs other than dolutegravir or raltegravir, regardless of the reason for switching regimens; switching of dose or formulation only was not considered a change of treatment line. Treatment line was described as “mixed” if a study included subjects on first‐ and subsequent‐line ART and data were not stratified.

Adverse events reported as grade 3 (severe) or grade 4 (life threatening or disabling) according to standard definitions [15] were recorded and could be due to clinical signs or symptoms, or laboratory findings. We report the number of subjects who experienced at least one grade 3 or 4 adverse event, rather than the total number of events. Grades 1 and 2 events were not extracted, nor were “serious adverse events,” unless the grade of event was reported.

Studies defined as cohort studies included prospective and retrospective observational studies and chart reviews. Studies are referred to by their name, where provided, and otherwise by first author and year of publication.

### 2.4 Evidence synthesis

For most outcomes, the proportion of subjects experiencing each outcome was presented. Scatterplots showing viral suppression were generated in Stata version 16, and safety outcomes were presented as heatmaps developed in Microsoft Excel.

### 2.5 Deviations from the protocol

Following publication of the protocol on PROSPERO, we added an additional risk of bias tool to assess non‐randomized interventional studies (discussed above). We also added an additional outcome of interest: changes in lipid variables (cholesterol and triglycerides) from baseline.

## 3 RESULTS AND DISCUSSION

### 3.1 Study selection

**Figure 1:**
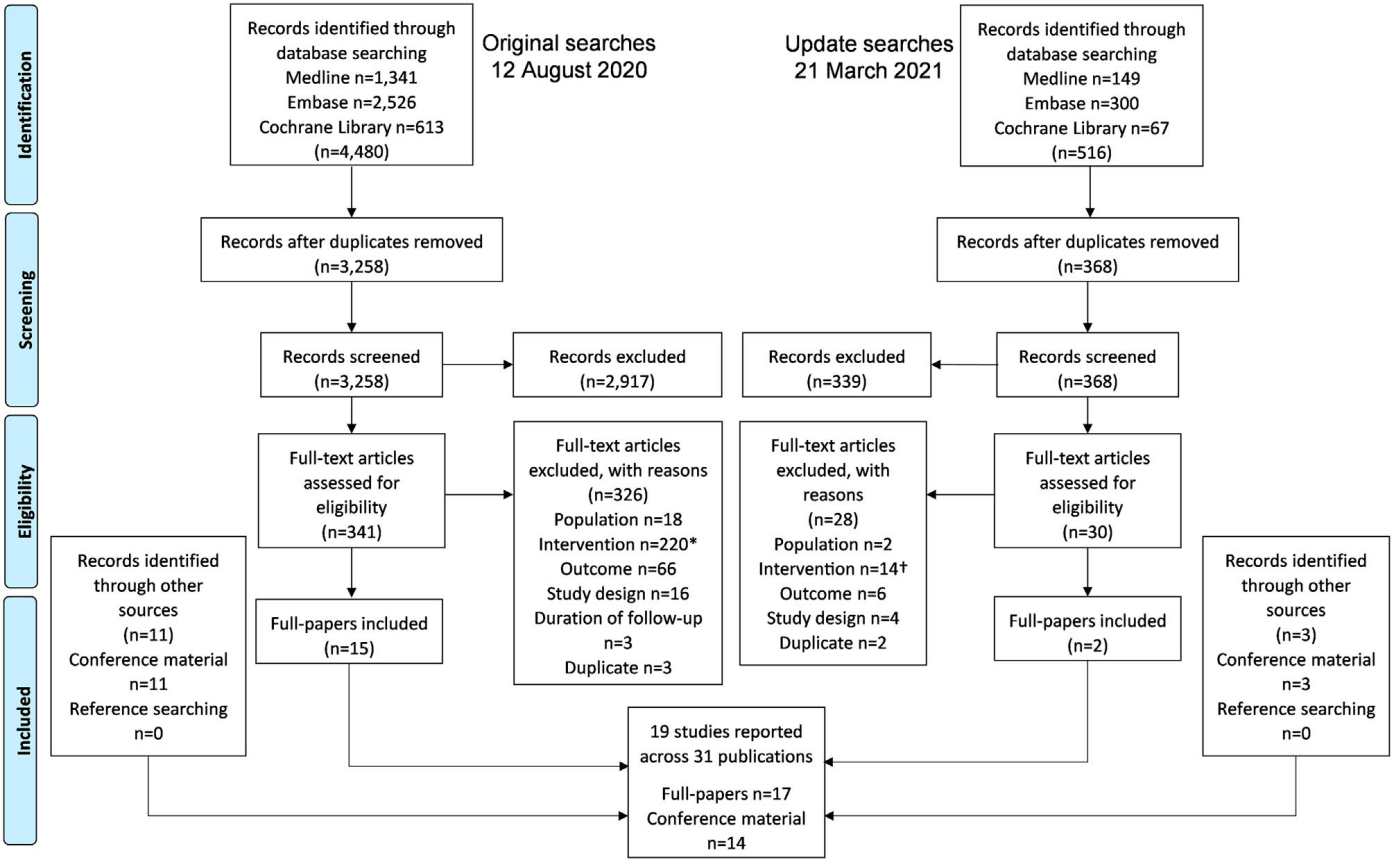
PRISMA flow diagram illustrating the number of included and excluded studies at each stage of the review. Note: The search strategies included terms for darunavir, lopinavir and tenofovir alafenamide (as well as dolutegravir and raltegravir); however, during full paper review, studies were categorized by the treatment of interest and only studies assessing dolutegravir and raltegravir are included here. ^*^ Of the 220 publications excluded because they assessed an intervention other than dolutegravir or raltegravir, two articles reported data on tenofovir alafenamide and 32 reported data on darunavir or lopinavir (solid formulations). † Of the 14 publications excluded because they assessed an intervention other than dolutegravir or raltegravir, six articles reported data on darunavir or lopinavir (solid formulations).

The searches for the review were conducted on 12 August 2020 and rerun on 21 March 2021 to capture studies published subsequently. Results of the searches and screening are presented in a PRISMA flow diagram (Figure 1). Overall, 3626 abstracts and 371 papers were screened. Nineteen studies relating to dolutegravir and/or raltegravir were included. These studies were published in 17 papers and 14 conference presentations; two studies reported data for both dolutegravir and raltegravir [16, 17]. Screening of trial registries identified 13 studies assessing dolutegravir and three assessing raltegravir that were either ongoing or completed, but with no published results identified in this review (File S2, Table S1).

### 3.2 Dolutegravir

#### 3.2.1 Study and subject characteristics

Eleven studies reported data on dolutegravir, with median follow‐up duration of 6–36 months: one RCT [18], one single‐arm trial [19] and nine observational studies [16, 17, 20, 21, 22, 23, 24, 25, 26, 27] (Table 3). Six studies recruited subjects from Europe only, three from sub‐Saharan Africa only and two from multiple geographic regions (Table 3). Of note, three studies included subjects being treated at the same hospital [20, 21, 22]; as the study time periods overlapped, some individuals may have been included in more than one study. In addition, we identified two sub‐studies of ODYSSEY assessing safety and pharmacokinetic outcomes in children and adolescents with and without tuberculosis [28, 29]. We included results from these sub‐studies if the studies reported safety outcomes not presented for the main trial.

**Table 3:** Study and baseline subject characteristics

Study name^a^	Country	Time period	Median^b^ duration of follow‐up (months)	*N*	Median age^c^ (IQR or range; years) [age category]^d^	Proportion receiving study drug as first‐line therapy	Viral suppression at study drug initiation?	Available outcome data
DOLUTEGRAVIR—RCT								
ODYSSEY [18, 28, 29, 48, 49]	Uganda, South Africa, Zimbabwe, Thailand, Portugal, Germany, Spain and the United Kingdom	Enrolled 2016–2018	33	Dolutegravir: 350 Standard of care: 357	Total study population: 12.2 (range 2.9, 18) [Children and adolescents]	44% (results stratified for some outcomes)	No	Treatment failure, CD4, mortality, grade 3/4 AE, drug‐related grade 3/4 AE, discontinuation due to AE, lipids
DOLUTEGRAVIR—SINGLE‐ARM TRIALS								
IMPAACT P1093 [19, 30, 31, 32, 33]	The United States and Thailand (children and adolescents); Brazil, Botswana, Kenya, South Africa, Tanzania, Uganda, Zimbabwe (children)	Infants and children <6 years: data to 30 April 2019 Adolescents: enrolled 2011–2012	Infants and children <6 years: 6 Adolescents: 35 (range 13, 45)	Infants and children <6 years: 51 Children 6–11 years: 38 Adolescents: 23	Results stratified by age group (total age range: 4 weeks to 17 years) [Infants, children and adolescents]	Infants and children <6 years: 29% Children and adolescents ≥6 years: 0%	No	VL, CD4, mortality, grade 3/4 AE, drug‐related grade 3/4 AE, discontinuation due to AE
DOLUTEGRAVIR—OBSERVATIONAL STUDIES								
Briand 2017 [20]	France	Began treatment 2014–2015	9 (IQR 5, 13)	50	18.0 (IQR 15.6, 20.2) [Adolescents]	2%	Mixed—some results stratified	VL, mortality, grade 3/4 AE, drug‐related grade 3/4 AE, discontinuation due to AE
ANRS EPF‐CO10 [21]	France	2016–2017	On treatment >6 months	69 on InSTI, of whom, 65 (94.2%) were on DTG	14.0 (IQR 7.3, 17.0) for subjects on InSTI [Children and adolescents]	Mixed (proportion unclear)	Not reported	VL
Frange 2019 [22]	France	Began treatment 2014–2017	Children: 16 (range 10, 43) Adolescents: 24 (range 9, 56)	84	Results stratified by age (5–11 years, 12–17 years) [Children and adolescents]	Children: 9% Adolescents: 12%	Mixed—some results stratified	VL, mortality, grade 3/4 AE, drug‐related grade 3/4 AE, discontinuation due to AE
Giacomet 2020 [23]	Italy	Not reported	Last follow‐up at 12 months	14	Mean age 16.1 years [Adolescents]	0%	Not reported	Lipids
Thivalapill 2020 [24, 50]	Eswatini	Transitioned to DTG 2019	Up to 12 months after switch	460	14 (IQR 12–16) [Children and adolescents]	0%	Yes	HAZ, WAZ
Bacha 2020 [25]	Tanzania	2019	Up to 10 months	681	Mean age 13.9 (range 5.0, 19.9) [Children and adolescents]	13%	Mixed—some results stratified	VL, discontinuation due to AE
Iyer 2021 [26]	Zambia	2019	Up to 36 months	211	Not reported but included individuals ≤18 years [Children and adolescents]	Not reported	Not reported	VL
DOLUTEGRAVIR AND RALTEGRAVIR—OBSERVATIONAL STUDIES								
Abo 2019 [16]	United Kingdom	Began treatment 2009–2018	DTG: 14 (IQR 6–27) RAL: 34 (IQR 6–54)	Dolutegravir: 29 Raltegravir: 21	Total study population: 15.0 (IQR 13.5, 16.4) [Children and adolescents]	Dolutegravir: 7% Raltegravir: 0%	No	VL, drug‐related grade 3/4 AE, discontinuation due to AE
CHIPS [17, 27]	United Kingdom and Ireland	2008–2019	DTG: 12 (IQR 5, 20)	Dolutegravir: 274	Dolutegravir: 14.9 (IQR 12.2 16.4)	Dolutegravir: 10%	Mixed—some results stratified	VL, CD4, grade 3/4 AE, drug‐related grade 3/4 AE
			RAL: 20 (IQR 5, 33)	Raltegravir: 99	Raltegravir: 12.9 (IQR 9.4, 15.0) [Children and adolescents]	Raltegravir: 14%Effectiveness results stratified by drug		
RALTEGRAVIR—RCT								
REALITY [34]	Zimbabwe, Uganda, Malawi, Kenya	2013–2015	11	Raltegravir‐intensified ART: 39 Standard ART: 33	Not reported for population <18 years [Children and adolescents]	100%	No	CD4, mortality
RALTEGRAVIR—SINGLE‐ARM TRIAL								
IMPAACT P1066 [35, 42, 43]	The United States of America, South Africa, Botswana, Brazil and Argentina	Enrolled 2007–2012; follow‐up to 2017	55	122	Results stratified by age group (total age range: 4 weeks to 19 years) [Infants, children and adolescents]	Infants 4 weeks to 6 months: 100% Older age groups: 0% Some results stratified	No	VL, CD4, mortality, grade 3/4 AE, drug‐related grade 3/4 AE, discontinuation due to AE
RALTEGRAVIR—OBSERVATIONAL STUDIES								
CoRISPe [38]	Spain	2007–2009	19 (IQR 11, 22)	19	16 (IQR 15, 18) [Children and adolescents]	0%	No	VL, CD4, mortality, drug‐related grade 3/4 AE, discontinuation due to AE
IeDEA global consortium [39]	Asia‐Pacific, Caribbean, Central and South America, East Africa, Southern Africa	Began treatment 2006–2017	24 (IQR 10, 36)	62	14.3 (IQR 11.2, 15.8) [Children and adolescents]	0%	No	VL, mortality, discontinuation due to AE
Ferreira 2019 [40]	Brazil	Switched to RAL 2017–2018	Up to 12 months after switch	221	7 (IQR 5, 10) [Children]	0%	Mixed	VL
Rozenszajn 2020 [37]	Argentina	Not reported	Viral subtype B: 13 Viral subtype F: 18	41	13.2 [Children and adolescents]	0%	No	VL
RALTEGRAVIR +/– DARUNAVIR—OBSERVATIONAL STUDIES								
Huerta‐Garcia 2016 [36]	Mexico	Not reported	11	16 on optimized regimen, of whom 13 on raltegravir	14.5 (IQR 11.2, 16.7) [Children and adolescents]	0%	No	VL, mortality, discontinuation due to AE
Thuret 2009 [41]	France	2006–2007	12 (range 9–21)	12	15 (range 12, 17) [Adolescents]	0%	No	VL, mortality, grade 3/4 AE, drug‐related grade 3/4 AE, discontinuation due to AE

Abbreviations: AE, adverse event; ANRS‐EPF, French Agency for Research on AIDS and Viral Hepatitis—French Perinatal Cohort; ART, antiretroviral therapy; CHIPS, Collaborative HIV Paediatric Study (United Kingdom and Ireland); CoRISPe, Cohort of the Spanish Paediatric HIV Network; DTG, dolutegravir; HAZ, height for age Z‐score; IeDEA, International epidemiology Databases to Evaluate AIDS; IMPAACT, International Maternal Pediatric Adolescent AIDS Clinical Trials; InSTI, integrase strand transfer inhibitor; IQR, interquartile range; RAL, raltegravir; RCT, randomized controlled trial; VL, viral load <50, <400, or <1000 copies/ml, as available in the publication; WAZ, weight for age Z‐score.

^a^ Where a study does not have a name, the first author and publication year are used.

^b^ Median duration of follow‐up unless otherwise specified.

^c^ Median age at baseline (start of study drug) unless otherwise specified.

^d^ Studies categorized into age groups (infant: <12 months; child: 1 to <12 years; and adolescent: 12 to <20 years) to simplify results presentation in scatterplots and heatmap.

Across the studies, 2330 children received dolutegravir‐based therapy (Table 3). Nine studies included children and adolescents, one study also reported data on infants [30] and two studies included adolescents only [20, 23]. Most studies included subjects in the target age range, but in Briand 2017, a quarter of subjects were over 20 years of age (median, 18.0 years, IQR 15.6–20.2) and were included within the results presented here [20]. Five studies included a small proportion of subjects (<15%) receiving dolutegravir as first‐line therapy, but none reported stratified safety data for first‐line treatment. One study included only subjects who were virally suppressed at baseline [24], four included a population with mixed viral load levels, three included subjects who were not suppressed and three did not report suppression at baseline (Table 3).

#### 3.2.2 Risk of bias

**Figure 2:**
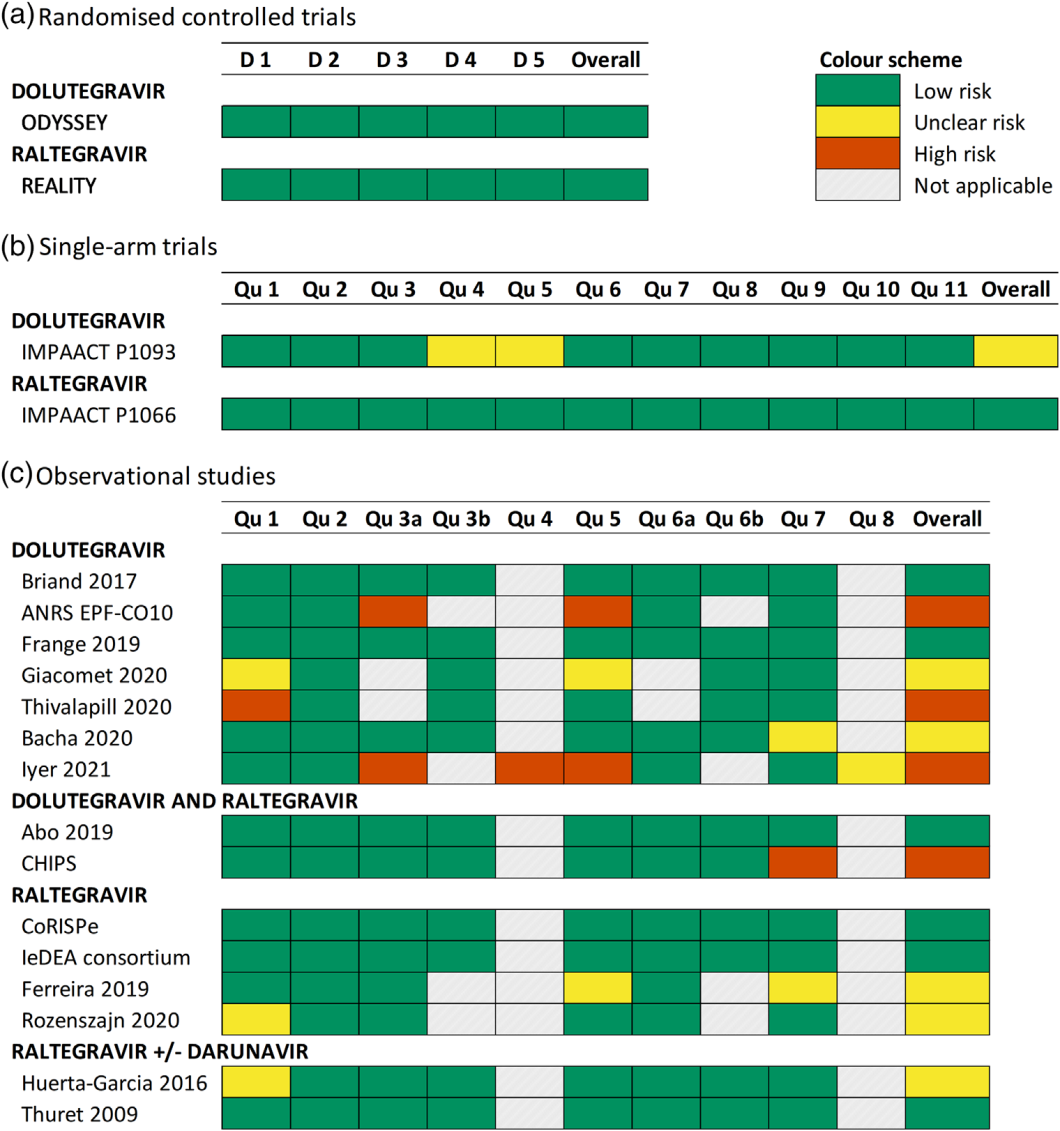
Summary of risk of bias assessment stratified by study design and treatment: (a) randomized controlled trials, (b) single‐arm trials and (c) observational studies. Green shading indicates low risk of bias, yellow shading indicates unclear risk, orange shading indicates high risk and grey indicates that the question was not applicable for that study. Note: For the assessment of single‐arm trials, none of the interventions were at group level, so Question 12 of the risk of bias tool was not applicable for all studies and is not presented here. List of assessment questions is provided in Table 2. Abbreviations: D, domain; Qu, question.

Results of the risk of bias assessment are shown in Figure 2, with additional details described in Table 4. Risk of bias for the ODYSSEY RCT was rated as low [18]. The single‐arm trial was deemed to be of unclear risk of bias (Figure 2 and Table 4) [19]. Of the nine observational studies, four were rated as high risk of bias [21, 24, 26, 27], two as unclear [20, 22] and three as low risk [21, 24, 26] (Figure 2 and Table 4).

**Table 4:** Summary of reasons for risk of bias classification for studies rated as unclear or high risk

Study name	Study design	Overall risk of bias classification	Reasons for risk of bias classification
DOLUTEGRAVIR			
IMPAACT P1093 [19, 30, 31, 32, 33]	Single‐arm trial	Unclear	Uncertainty around whether all eligible participants were enrolled and whether the study was sufficiently powered; no sample size calculation reported.
ANRS EPF‐CO10 [21]	Observational study	High	Study was not designed to assess dolutegravir alone; viral load and other key prognostic factors not reported at baseline.
Giacomet 2020 [23]	Observational study	Unclear	Conference poster with limited methodological data provided.
Thivalapill 2020 [24, 50]	Observational study	High	Study excluded subjects who had viral load >200 copies/ml at any time during the study; although study only reported safety data, it is possible that key safety events were missed.
Bacha 2020 [25]	Observational study	Unclear	Conference poster—median duration of follow‐up not provided but study took place over 10 months, so median time on treatment may be less than 6 months.
Iyer 2021 [26]	Observational study	High	Conference presentation assessing multiple interventions, limited background characteristics.
RALTEGRAVIR			
Ferreira 2019 [40]	Observational study	Unclear	Conference poster with limited baseline data provided. Median follow‐up not reported; follow‐up varied from 30 to 365 days, so median time on treatment may have been less than 6 months.
Rozenszajn 2020 [37]	Observational study	Unclear	Conference poster with limited details on selection of the study population.
DOLUTEGRAVIR AND RALTEGRAVIR			
CHIPS [17, 27]	Observational study	High	Conference poster—proportion of participants with outcome data varied from 30% to 65%.
RALTEGRAVIR +/– DARUNAVIR			
Huerta‐Garcia 2016 [36]	Observational study	Unclear	Unclear if all children within a particular time period were included.

#### 3.2.3 Efficacy and effectiveness outcomes

**Figure 3:**
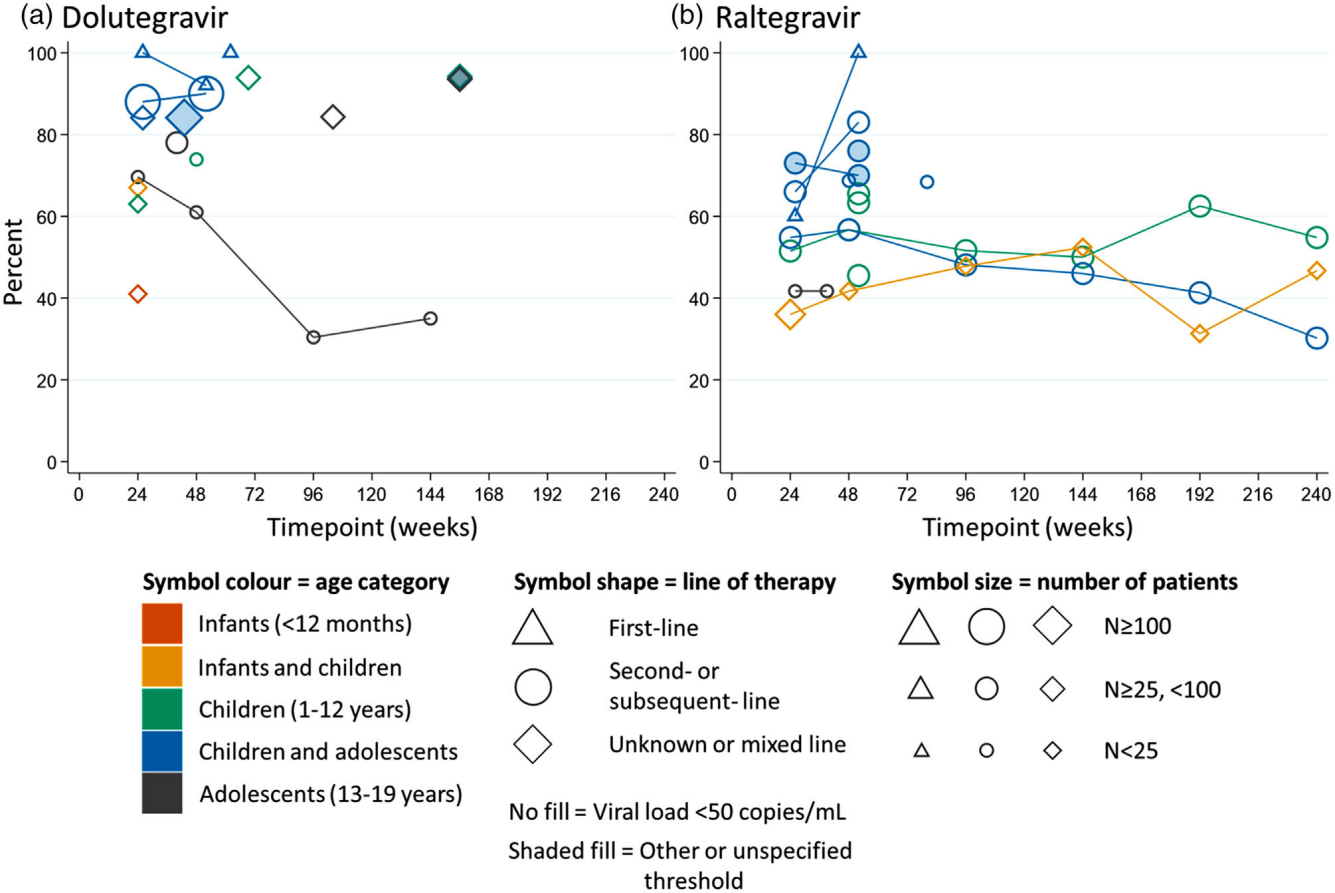
Proportion of infants, children and adolescents achieving viral suppression of <50 copies/ml (open symbols), or <400 copies/ml, <1000 copies/ml or viral suppression at an unspecified threshold if <50 copies/ml was not reported (shaded symbols) after treatment with (a) dolutegravir or (b) raltegravir over time. Results are stratified by age (colour of symbol), line of therapy (shape of symbol) and study size (size of symbol). Connecting lines link data follow‐up points from the same study population. Data from an individual study may be presented at more than one timepoint; however, at a given timepoint, data from a group of subjects are only presented once (overall data were prioritized over sub‐group data). For randomized controlled trials and single‐arm studies, the timepoint represents the duration of follow‐up; for observational studies, the timepoint represents the median follow‐up time of the study. A table showing the viral load data used to create the scatterplot is included in File S2 (Table S2).

Viral load data were reported in eight studies, which included a total of 1281 subjects (Figure 3a; File S2, Table S2): one single‐arm trial [19, 30, 31, 32, 33] and seven observational studies [16, 17, 20, 21, 22, 25, 26, 27] (Table 3). Most of the studies reported virological outcomes through 6–12 months after dolutegravir initiation; three studies reported extended follow‐up (Table 3) [22, 26, 31]. Two observational studies assessed first‐line dolutegravir‐based therapy in children and adolescents [16, 17]. Both reported that >90% of subjects achieved viral suppression, but populations were small (*n* = 2, 62 weeks follow‐up [16]; *n* = 14, 26 and 52 weeks follow‐up [17]), and one study was rated high risk of bias [17], so results should be interpreted with caution. Three studies reported data on subjects receiving dolutegravir as second‐ or subsequent‐line therapy [17, 19, 20, 31, 32, 33]. One study, IMPAACT P1093, reported viral suppression of <70% in this population at 24–144 weeks (*n* = 23); however, this study recruited adolescents on a failing regimen who were switching to dolutegravir (baseline viral load >1000 copies/ml) after a median of 12.5 years of prior treatment; in addition, many participants were reported to have psychosocial or behavioural challenges, which may have affected outcomes [19, 31].

Five studies reported data on populations with mixed or unknown treatment history [21, 22, 25, 26, 30]. The single‐arm study IMPAACT P1093, in subjects who were unsuppressed at baseline, reported poor suppression at 24 weeks (7/17, 41%) in infants aged 4 weeks to 6 months, compared with infants and children aged 6 months to <2 years (6/9, 67%) and children aged 2–6 years (5/8, 63%) [30]. The four observational studies reported high rates of viral suppression, ranging from 84% (58/69 in ANRS EPF‐CO10 and 499/593 in Bacha 2020) to 94% (number of events not reported in Iyer 2021) [21, 22, 25, 26]. Although ANRS EPF‐CO10 and Iyer 2021 were rated as high risk of bias, the estimates for viral suppression from the two studies were similar to Frange 2019 and Bacha 2020. ODYSSEY reported treatment failure rather than viral suppression (File S2, Table S3) [18]. After 96 weeks, 15 subjects receiving first‐line dolutegravir‐based therapy experienced treatment failure compared with 34 on standard of care (estimated proportions: 10% vs. 23%, difference = –12.5%, *p* = 0.003). For subjects receiving second‐line dolutegravir‐based therapy, 32 experienced treatment failure compared with 41 on standard of care (estimated proportions: 17% vs. 21%, difference = –4.6%, *p* = 0.22).

CD4 data were reported inconsistently across the studies assessing dolutegravir; three studies reported mean/median change from baseline (File S2, Tables S4 and S5) [18, 19, 27, 30, 31, 32, 33]. After 24 weeks of follow‐up, median change in CD4 percent was between +3% and +5% in three sub‐groups of children aged <6 years [30]. After 48 weeks of follow‐up in populations aged ≥6 years, median change in CD4 cell count ranged from +39 cells/μl (IQR –97, +136) in 52 adolescents virally suppressed at baseline on second‐ or subsequent‐line of therapy [17], to +387 cells/μl (IQR +49, +575) in 23 children aged 6 to <12 years virally unsuppressed at baseline on second‐ or subsequent‐line of therapy [19].

#### 3.2.4 Safety outcomes

##### 3.2.4.1 Mortality

**Figure 4:**
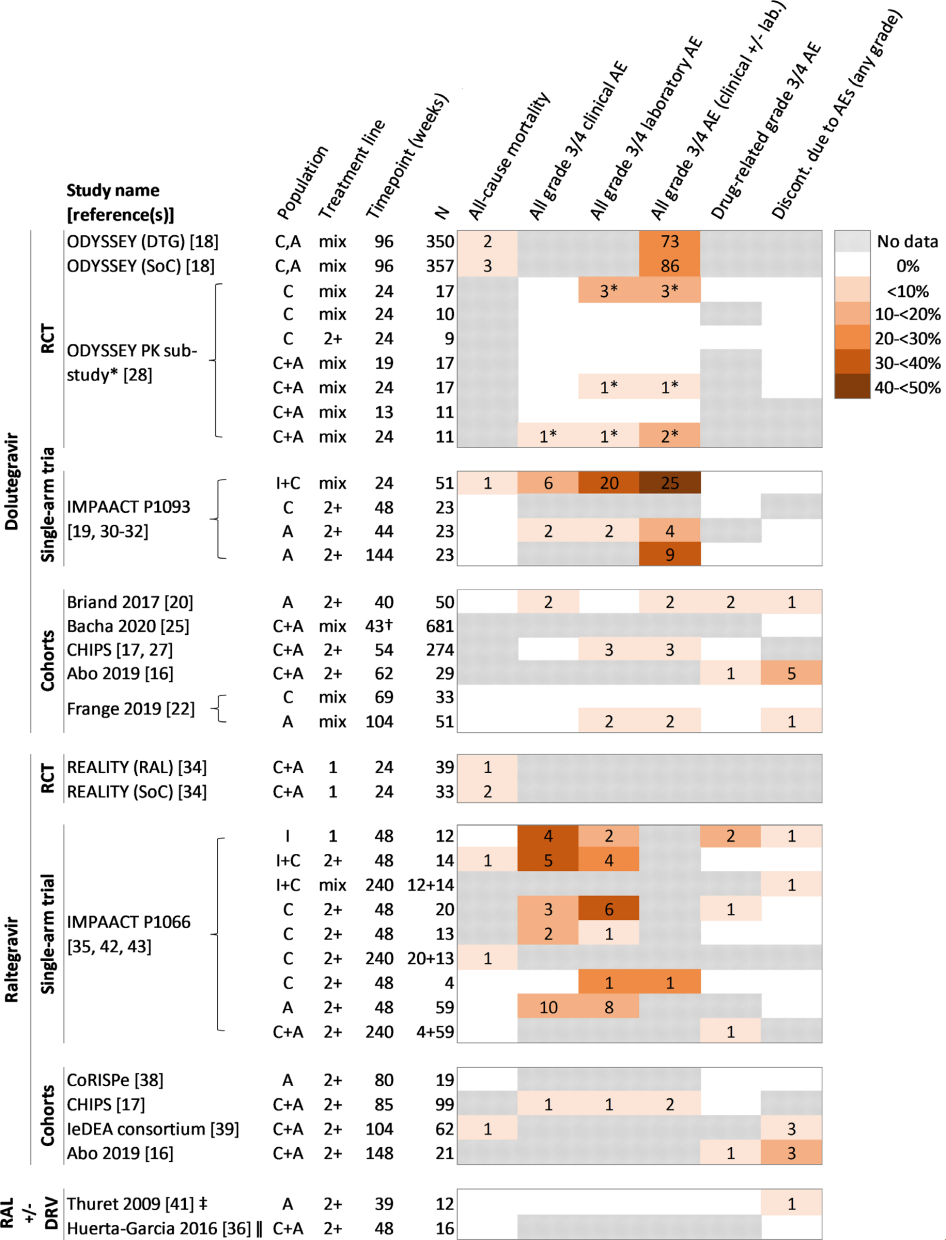
Proportion of subjects exposed to dolutegravir or raltegravir with reported safety outcomes, ordered by study, sub‐population (age or weight group) and timepoint (median follow‐up time for observational studies and ODYSSEY pharmacokinetic sub‐study; duration of follow‐up for other studies). Multiple rows for the same study correspond to different sub‐populations and/or timepoints (further details are provided in File S2, Table S6); subjects or events may, therefore, appear in more than one row. Numbers in coloured cells correspond to the number of subjects who experienced events. Where data are reported for grade 3/4 clinical adverse events and for grade 3/4 laboratory adverse events but not for all grade 3/4 adverse events, this is because overlap between clinical and laboratory events was unclear or not reported. ^*^ Subjects in the ODYSSEY pharmacokinetic sub‐study were also included in the main trial; results are presented for the sub‐study where additional outcomes were reported to those presented for the main trial; † follow‐up duration was “up to 43 weeks”; ‡ all children were on raltegravir, ritonavir‐boosted darunavir and etravirine; ǁ 13 of the 16 children were on raltegravir, 10 of whom also received darunavir. Abbreviations: 1, first‐line; 2+, second‐ or subsequent‐line; A, adolescents; AE, adverse event; C, children; CHIPS, Collaborative HIV Paediatric Study (United Kingdom and Ireland); CoRISPe, Cohort of the Spanish Paediatric HIV Network; discont., discontinuation; DRV, darunavir; DTG, dolutegravir; Gr 3/4 AE, grade 3 or 4 adverse event; I, infants; IeDEA, International epidemiology Databases to Evaluate AIDS; IMPAACT, International Maternal Pediatric Adolescent AIDS Clinical Trials; mix, mixed or unspecified line of treatment; PK, pharmacokinetics; RAL, raltegravir arm; SoC, standard of care arm.

Four studies reported all‐cause mortality in subjects on dolutegravir [18, 19, 20, 22, 30, 31, 32] (Figure 4). In ODYSSEY, there were two deaths (0.6%) in the dolutegravir arm, and three (0.8%) in the standard of care arm over median follow‐up of 142 weeks [18]. One of the deaths in the dolutegravir arm was due to tuberculosis‐related causes [29]. In IMPAACT P1093, one child (2%) in a population of infants and children (4 weeks to 6 years; *n* = 51) died of acute gastroenteritis dehydration during 24 weeks of follow‐up (not considered related to dolutegravir) [30]. No deaths were reported in the remaining two cohort studies [20, 22].

##### 3.2.4.2 Grade 3/4 adverse events

Five studies reported on the overall proportion of subjects with grade 3/4 adverse events (clinical and laboratory) [18, 19, 20, 22, 27, 30, 31], which ranged from 0% (0/33) in children with median follow‐up of 69 weeks in Frange 2019 [22], to 49% (25/51) in infants and children followed up for 24 weeks in IMPAACT P1093, all of whom had HIV viral loads >1000 copies/ml at baseline [30] (Figure 4). In ODYSSEY, 73 subjects (21%) in the dolutegravir arm experienced grade 3/4 clinical or laboratory events compared with 86 subjects (24%) in the standard of care arm (*p* = 0.30) [18]. The ODYSSEY pharmacokinetic sub‐study reported the breakdown of grade 3/4 clinical and laboratory events: among 62 children and adolescents included in the sub‐study, there was one clinical event and five laboratory events [28]. In one study, total number of events was reported, but number of subjects was unclear: seven events were reported in 23 children aged 6 to <12 years in IMPAACT P1093 [32] (data not included in Figure 4). Across the studies, three grade 3/4 neurological or psychiatric events were reported: two in Briand 2017 (Table 5) and one in IMPAACT P1093 (depression and attempted suicide in a subject with a pre‐existing mental disorder) [31].

**Table 5:** Details of drug‐related grade 3/4 adverse events reported across the studies of dolutegravir and raltegravir

Study name	Drug	Age group	Drug‐related grade 3/4 adverse events	Neurological or psychiatric event
Briand 2017 [20]	Dolutegravir	Adolescent	Moderate headache and dizziness (treatment continued)	Yes
		Adolescent	Severe dizziness, sleeping disorders and anxiety (resolved after dolutegravir interruption)	Yes
Abo 2019 [16]	Dolutegravir, raltegravir	Child	Raised creatine kinase, thought to be treatment related but subsequently considered potentially related to a hereditary condition (child exposed to dolutegravir and raltegravir)	No
IMPAACT P1066 [35, 42, 43]	Raltegravir	Infant	Serious erythematous rash leading to permanent discontinuation of raltegravir (subject also diagnosed with pneumocystis jirovecii pneumonia)	No
		Infant	Immune reconstitution syndrome at the site of neonatal Bacillus Calmette–Guérin immunization, which did not lead to treatment discontinuation	No
		Child	Increased alanine aminotransferase and aspartate aminotransferase	No
		Adolescent	Psychomotor hyperactivity, abnormal behaviour and insomnia	Yes

Three grade 3/4 events were considered possibly related to dolutegravir in two of six studies reporting drug‐related grade 3/4 adverse events (Figure 4 and Table 5) [16, 17, 20, 22, 27, 28, 30, 31, 32]. Two were reported in Briand 2017 [20] and one in Abo 2019, which did not report overall grade 3/4 adverse events (Table 5) [16].

##### 3.2.4.3 Discontinuations due to adverse events

Data on discontinuations for adverse events of any grade were reported in six studies (Table 3). No events were observed in three studies (follow‐up to 144 weeks in IMPAACT P1093, 24 weeks in ODYSSEY pharmacokinetic sub‐study and up to 43 weeks in Bacha 2020) [19, 25, 28, 30, 31] (Figure 4). One discontinuation (2%) due to a grade 3/4 adverse event was reported during a median of 40 weeks of treatment in Briand 2017 (Table 5), while one adolescent (2%) discontinued treatment due to dizziness and sleep disturbance (grade not reported) in Frange 2019 [20, 22]. In Abo 2019, five subjects (17%) discontinued dolutegravir over a median of 62 weeks due to adverse events (mood deterioration, sleep disturbance and raised creatine kinase, raised gamma‐glutamyl transferase, abdominal pain and hypertension; grades not reported) [16].

##### 3.2.4.4 Other outcomes

Limited data on growth and lipid levels were reported. Thivalapill 2020 reported a mean height for age Z‐score (HAZ) of –0.1 (95% CI –0.1, 0) after more than 26 weeks of follow‐up among 460 adolescents on dolutegravir [24]. The HAZ rate change was –0.1 (95% CI –0.1, 0; *p*<0.01) per year after dolutegravir initiation, while the rate of change in weight for age Z‐score (WAZ) was 0.2 per year (95% CI 0.2, 0.3; *p*<0.001); however, subjects were only followed up for up to 1 year, and the study was considered high risk of bias.

Total cholesterol data in children and adolescents were reported in ODYSSEY, comparing dolutegravir (*n* = 350) to standard of care (*n* = 357; 92% of subjects on first‐line therapy received efavirenz, and among those on second‐line therapy, 72% received ritonavir‐boosted lopinavir and 25% ritonavir‐boosted atazanavir) [18]. After 48 weeks of treatment, total cholesterol decreased with dolutegravir treatment (mean change from baseline: –5 mg/dl, 95% CI –8, –3) and increased with standard of care (+10 mg/dl, 95% CI +7, +13). After 144 weeks, mean change was –2 mg/dl (95% CI –7, +2) in the dolutegravir arm and +11 mg/dl (95% CI +6, +16) in the standard of care arm. Similarly, in Giacomet 2020, mean total cholesterol decreased from 190 mg/dl at baseline to 168 mg/dl in 14 adolescents on dolutegravir for 52 weeks (*p* = 0.0057) [23]. A significant difference was also reported for low‐density lipoprotein cholesterol (decrease from 109 to 96 mg/dl; *p* = 0.025), but the change was not significant for high‐density lipoprotein.

### 3.3 Raltegravir

#### 3.3.1 Study and subject characteristics

Ten studies of children and adolescents prescribed raltegravir were identified, with median follow‐up duration ranging from 11 to 55 months: one RCT (REALITY) [34], one single‐arm trial (IMPAACT P1066) [35] and eight observational studies [16, 17, 36, 37, 38, 39, 40, 41] (Table 3). Two studies reported outcome data on children and adolescents receiving raltegravir in combination with darunavir [36, 41]. Four studies were set only in Europe [16, 17, 38, 41], one only in sub‐Saharan Africa [34], three in Central or South America [36, 37, 40] and two in multiple geographic regions [35, 39].

Across the studies, 649 children received raltegravir‐based therapy (Table 3). Eight studies included both children and adolescents, and one study also included infants (IMPAACT P1066) [42, 43]. One study (REALITY) included exclusively subjects on first‐line therapy, all of whom had CD4 counts <100 cells/μl at baseline [34]. Of note, four studies included populations where most were heavily pre‐treated (median of ≥3 lines of previous therapy) at start of raltegravir [35, 36, 39, 41, 43], while in a fifth study, raltegravir was used as part of a compassionate use programme for children who were virological non‐responders on previous regimens [38]. Eight studies included only subjects who were unsuppressed at baseline (Table 3).

#### 3.3.2 Risk of bias

The REALITY RCT and the single‐arm trial, IMPAACT P1066, were classified as low risk of bias across all domains of the relevant risk of bias tools (Figure 2) [34, 35]. Among observational studies, CHIPS was deemed high risk of bias (Table 4) [17].

#### 3.3.3 Efficacy and effectiveness outcomes

Eight studies reported viral suppression data on 554 children (stratified into 13 sub‐populations) receiving raltegravir (Figure 3b; File S2, Table S2)—one single‐arm trial and seven observational studies (Table 3) [17, 35, 36, 37, 38, 39, 40, 41]. There were limited data on raltegravir‐based first‐line therapy; in four children and adolescents in CHIPS [17], all achieved viral suppression at 48 weeks, while in infants with vertical transmission despite maternal/infant prophylaxis who had HIV RNA ≥1000 copies/ml at baseline (IMPAACT P1066), there was poor suppression (3/9, 33% at 24 weeks) [42]. However, both studies were small, and these data should be interpreted with caution. The infant data from IMPAACT P1066 are not presented in Figure 3b; instead, long‐term follow‐up of this population in combination with children is presented [43].

Most of the raltegravir data were for populations on second‐ or subsequent‐line of therapy: one single‐arm trial [35, 42, 43] and seven observational studies [17, 36, 37, 38, 39, 40, 41]. At 1 year follow‐up, viral suppression ranged from 42% (5/12) to 83% (44/53) [17, 41]. In IMPAACT P1066, viral suppression at 240 weeks was 30% (13/43) in children (2 to <12 years) and 55% (17/31) in children and adolescents (6–19 years) who were not virally suppressed at baseline [43]. The proportion of infants and children (4 weeks to 2 years old) achieving viral suppression during long‐term follow‐up in IMPAACT P1066 was relatively stable, with 42% (10/24) of infants/children suppressed at 48 weeks compared with 47% (7/15) at 240 weeks [43]. However, data were not available for all children at all timepoints.

Three studies provided data on mean or median change in CD4 levels in subjects treated with raltegravir (File S2, Tables S4 and S5) [17, 34, 35, 42, 43]. After 48 weeks follow‐up in populations <6 years old, mean change in CD4 percent ranged from +4.3% (95% CI 1, 7.6) in 20 children aged 2 to <6 years [35] to +8.7% (95% CI 2.7, 14.8) in 12 infants who previously received unsuccessful prophylaxis for vertical transmission [42]; both populations were from IMPAACT P1066. In populations ≥6 years of age on first‐line raltegravir‐based therapy after 48 weeks of follow‐up, change in CD4 cell counts was a median of +256 cells/μl (95% CI –69, +594) in four children and adolescents in CHIPS [17] and a mean of +323 cells/μl (95% CI 255.3, 390.2) in 39 children and adolescents in REALITY [34]. In subjects aged ≥6 years on second‐ or subsequent‐line of therapy, change in CD4 cell count from baseline ranged from a median of –121 cells/μl (IQR –375, +170) in 13 children and adolescents in CHIPS who were suppressed at baseline, to a median of +237 cells/μl (95% CI +60, +351) in 26 children and adolescents in CHIPS who were unsuppressed at baseline [17].

#### 3.3.4 Safety outcomes

##### 3.3.4.1 Mortality

Six studies (one RCT, one single‐arm trial and four observational studies) reported on mortality in subjects on raltegravir (Figure 4) [34, 35, 36, 38, 39, 41, 42, 43]. The REALITY trial reported mortality data for children and adolescents on a first‐line raltegravir‐intensified regimen (nevirapine or efavirenz‐based regimen, plus raltegravir) for 12 weeks with 24 weeks of follow‐up [34]. There was one death (3%) in the raltegravir‐intensified ART arm compared with two (6%) in the standard ART arm (nevirapine or efavirenz‐based regimen) (hazard ratio 0.43, 95% CI 0.04, 4.70, *p* = 0.74). Two deaths in subjects on second‐ or subsequent‐line raltegravir were reported in IMPAACT P1066 (Figure 4) [42, 43]. In the cohort of infants and children (6 months to <2 years; *n* = 14) followed up for 48 weeks in IMPAACT P1066, one child with multiple co‐morbidities died of gastroenteritis, but this was not considered related to raltegravir [42]. In children aged 2–12 years (*n* = 33) receiving raltegravir chewable tablets, one child died of gastroenteritis at week 59 of 240 weeks of follow‐up [43]. In addition, a teenage girl died from pneumonia in IMPAACT P1066, but the death was not included in the final analysis, as the subject was not on the final selected dose of raltegravir. One death (2%) was reported in a child in the IeDEA global consortium (*n* = 62) during 104 weeks of follow‐up [39].

##### 3.3.4.2 Grade 3/4 adverse events

Three studies (one single‐arm trial and two observational studies) reported on clinical and laboratory grade 3/4 adverse events (Figure 4) [17, 35, 41, 42, 43]. In 12 treatment‐naïve infants receiving raltegravir granules in IMPAACT P1066, four infants (33%) experienced a grade 3/4 clinical event, while two infants (17%) experienced a grade 3/4 laboratory event by week 48 (overlap unclear) [42]. Grade 3/4 adverse events were also reported across five populations on second‐ or subsequent‐line of therapy in IMPAACT P1066 (overlap unclear) [30, 35, 42, 43]. In infants and children on raltegravir granules (*n* = 14) for 48 weeks, 38% (*n* = 5) and 29% (*n* = 4) experienced clinical and laboratory grade 3/4 adverse events, respectively [42]. Across the four arms of the study assessing children or adolescents at 48 weeks, clinical grade 3/4 adverse events occurred in 0–17% and laboratory events in 8–30% of children (Figure 4) [35, 43]. Grade 3 or 4 adverse events were also reported in two subjects (2%) on raltegravir in CHIPS after 85 weeks of follow‐up [17]. Thuret 2009 reported no grade 3/4 adverse events in 12 adolescents treated with raltegravir, etravirine and darunavir/ritonavir for 39 weeks [41].

Two of the four clinical events reported in treatment‐naïve infants receiving raltegravir granules in IMPAACT P1066 were considered possibly, probably or definitely related to raltegravir (Table 5) [42]. In addition, two subjects in IMPAACT P1066 [35, 43] and one in Abo 2019 [16] experienced grade 3/4 adverse events considered possibly related to study drug (latter subject also exposed to dolutegravir). One subject across the raltegravir studies experienced a grade 3/4 neurological or psychiatric event (Table 5) [43].

##### 3.3.4.3 Discontinuations due to adverse events

Six studies reported data on discontinuations due to adverse events (any grade), which occurred in less than 10% (and often 0%) of children and adolescents on raltegravir in nearly all of these studies (Figure 4) [16, 35, 36, 38, 39, 41, 42, 43]. The only exception was Abo 2019, in which three subjects (14%) discontinued raltegravir due to adverse events (raised creatine kinase, neutropenia and suspected allergic reaction; grades not reported) over a median of 148 weeks of treatment; two events were subsequently found to have alternative causes, and the other resolved upon raltegravir cessation [16].

##### 3.3.4.4 Other outcomes

Only two studies reported data on growth or lipid levels in subjects on raltegravir. Huerta‐Garcia 2016 reported no change in median HAZ from baseline (–2.05, IQR –2.87, –1.13) to 48 weeks (–1.75, IQR –2.72, –1.41; *p* = 0.187) among 16 heavily pre‐treated children and adolescents on an optimized regimen (13/16 on raltegravir) [36]. Median WAZ after 48 weeks was –1.46 (IQR –2.05, –0.81) and was not significantly different from baseline (–1.66, IQR –2.24, –0.80, *p* = 0.932). Thuret 2009 reported that median WAZ in an adolescent population treated for 39 weeks increased significantly to –0.68 (range –2.48, 1.4) from a baseline of –1.18 (range –2.63, 1.4, *p* = 0.033) [41].

### 3.4 Summary of findings and implications for research

Findings from this systematic review suggest that dolutegravir and raltegravir are safe and effective in infants, children and adolescents living with HIV. Across dolutegravir studies, efficacy/effectiveness was high, with only three datapoints (of 21 datapoints from eight studies) showing viral suppression in less than 60% of the study population; all three were studies with small sample sizes (<25). However, there were limited data in infants and young children and few data on long‐term effectiveness beyond 2 years. The majority of data were from studies assessing second‐, subsequent‐ or mixed‐lines of therapy, although initial, limited data suggest that dolutegravir is effective for first‐line treatment. Although viral suppression data from ODYSSEY were not available at the time of this review, this trial has since published results through 96 weeks. For first‐line treatment, viral load <50 copies/ml was reported in 80% (117/146) of participants in the dolutegravir arm and 81% (113/140) in the standard of care arm, while for second‐line treatment, 81% (153/196) of participants in the dolutegravir arm and 72% (139/200) in the standard of care arm were virally suppressed at 96 weeks [44]. In most raltegravir studies, over 50% of subjects achieved viral suppression at 24 and 48 weeks. In long‐term follow‐up (≥144 weeks), the proportion of children and adolescents with viral suppression ranged from 30% to 63%. However, there were limited data on raltegravir as first‐line therapy and limited data in infants. Importantly, five of seven raltegravir studies reporting viral suppression data included heavily pre‐treated subjects, which may explain why lower proportions of subjects achieved viral suppression compared with studies of dolutegravir. As raltegravir is now recommended in infants and young children as an alternative first‐line treatment, opportunities to monitor effectiveness and safety in this population group should be sought.

Few deaths were reported across the studies on dolutegravir and raltegravir, and none were due to adverse events related to either drug. Although the proportion of subjects with clinical and/or laboratory grade 3/4 adverse events ranged from 0% to nearly 50% across the studies, few events were considered drug related (<10%), and there were few discontinuations for adverse events of any grade (range 0–17%). The highest proportions of grade 3/4 adverse events were reported in the IMPAACT P1066 and IMPAACT P1093 trials, which recruited subjects with high viral loads, many of whom had extensive treatment experience. This review also highlighted the need for further safety data in infants, who were included only in registrational trials [30, 42]. ODYSSEY has since presented data showing no safety concerns for children weighing <14 kg treated with dolutegravir (median follow‐up 120 weeks) [45]. Two single‐arm trials assessing raltegravir in infant populations were identified through searches of trial registries, with planned completion dates in 2019 and 2031 (IMPAACT P1115/NCT02140255 and IMPAACT P1101/NCT01751568). There were also limited data on weight gain in children and adolescents on dolutegravir or raltegravir, although the ODYSSEY trial recently published additional data, showing improved growth with no excess weight gain in children and adolescents on dolutegravir, compared with standard of care [46]. The randomized trial, SMILE, identified via trial registry searches, pre‐specified weight as an outcome and also presented data recently, which showed significant increases in weight and body mass index at 48 weeks in children on an integrase inhibitor plus boosted darunavir compared with standard of care [47].

### 3.5 Strengths and limitations

To our knowledge, this is the first systematic review on efficacy/effectiveness and safety of dolutegravir and raltegravir in infants, children and adolescents. Although we did not conduct any meta‐analyses, this narrative review summarizes the current evidence‐base and provides reassurance to support the expanding rollout of dolutegravir‐based regimens. Many of the studies identified had relatively small sample sizes, of less than 100 subjects, which might explain some of the variability in the proportions of subjects experiencing adverse outcomes. Variability could also be explained by differences in reporting of adverse events between observational studies and clinical trials; furthermore, not all studies clearly stated the grading standards used for recording adverse events. For pragmatic reasons, we restricted our searches to English and French publications, but we recognize that this could cause language bias in the results of our review.

Many of the studies identified were observational and, therefore, have more potential sources of bias than RCTs or single‐arm studies. In this review, four of the 15 observational studies were rated high risk of bias and five as unclear risk. However, such studies remain important in providing real‐world data from different clinical settings. Data on longer‐term outcomes of these new drugs and formulations are critical. This review identified a wide range of studies with disparate research questions. Some studies reported outcomes which may have been of clinical or scientific interest but are not reported here as they were not comparable to the results of other studies.

## 4 CONCLUSIONS

In this review, dolutegravir and raltegravir were found to be safe and effective across the studies identified. These findings support the current WHO treatment guidelines, which recommend a dolutegravir‐based regimen for first‐line treatment of HIV in children and adolescents, and a raltegravir‐based regimen in neonates for whom dolutegravir dosing is not yet approved [5]. However, long‐term monitoring of the use of integrase inhibitors in paediatric populations will be important to fill evidence gaps, particularly for adverse outcomes reported in adult studies, including metabolic and neuropsychiatric outcomes, and in relation to their use in infants, and in first‐line regimens.

## COMPETING INTERESTS

AJ and IJC were authors on the two Collaborative HIV Paediatric Study (CHIPS) publications included in the review [17,27]. AJ and IJC report recent grant funding from ViiV Healthcare/GSK, which is the marketing authorization holder for Tivicay (dolutegravir). There were no other competing interests.

## AUTHORS’ CONTRIBUTIONS

CLT, JOR, EM, IJC, AJ, MV, JJ, VL, FR and MP contributed to developing the study protocol. JOR conducted the searches. JOR, CLT and EM contributed to screening of abstracts and papers, extracted the data from selected studies and assessed the studies for risk of bias. CLT and JOR drafted the paper. JOR, CLT and HC developed the figures. All authors reviewed the manuscript and approved the final draft.

## FUNDING

This work was supported by the World Health Organization and the International AIDS Society's Collaborative Initiative for Paediatric HIV Education and Research (CIPHER) Prospero number CRD42020204432.

## DISCLAIMER

The opinions expressed here are those of the authors and do not necessarily reflect the views of the funder. All authors had full access to all the data in the study, and the corresponding author had final responsibility for the decision to submit for publication.

## Supporting information


**File S1**. Details of data sources, search strategies and data extracted.

**File S2**. Supplementary tables.
**Table S1**. Study characteristics of clinical trials on dolutegravir or raltegravir in infants, children and/or adolescents identified through searching of registries, March 2021, for which no publications were identified at the time of the searches.
**Table S2**. Viral load data used to create the scatterplots presented in the paper (Figure 3).
**Table S3**. Treatment failure data from ODYSSEY.
**Table S4**. CD4 cell count or percent at baseline in studies in which data on change in CD4 measurement from baseline were also reported.
**Table S5**. Change in mean or median CD4 cell count or CD4 percent from baseline.
**Table S6**. Details of population sub‐groups presented in Figure 4 (age‐bands, weight groups and dose/formulations).

## Data Availability

The data extracted for this systematic review are available within the article and its supplementary materials.
