# Supplementary material for: Effectiveness and safety of dolutegravir and raltegravir for treating children and adolescents living with HIV: a systematic review
Source: J Int AIDS Soc. 2022 Nov 14;25(11):e25970. doi: 10.1002/jia2.25970 (PMC9663860; doi:10.1002/jia2.25970)
Supplement: Supplementary file 1 — File S1. Details of data sources, search strategies and data extracted. [file JIA2-25-e25970-s001.docx]

**Supporting file 1**

**A1. Data sources and searches**

The following electronic databases were searched: MEDLINE, MEDLINE In-Process, MEDLINE E-pub ahead of print (via Ovid), Embase (via Ovid), and The Cochrane Library using both free-text terms and index terms (Section A2). The searches combined terms for HIV, the interventions of interest, and terms for children and adolescents. Searches filtered out publication types that were not of interest (e.g. narrative reviews). In addition, the following grey literature sources were searched to limit the impact of publication bias on the results of the review:

- Clinical trial registries were searched for studies that were updated since January 2018 to capture trials that were ongoing and may or may not have already published outputs (search strategy shown in Section A2):
  - ClinicalTrials.gov
  - WHO International Clinical Trials Registry Platform (<http://apps.who.int/trialsearch/>)
  - EudraCT (www.clinicaltrialsregister.eu)
- References from relevant sections of the most recent version of international guidelines on HIV treatment:
  - WHO 2016 ARV guidelines [1]
  - US National Institutes of Health Guidelines for the Use of Antiretroviral Agents in Pediatric HIV Infection 2020 [2]
  - Penta 2015 and 2016 guidelines [3, 4] and the 2019 update [5]
- Conference abstract books were searched for each of the relevant drug names and the most common abbreviation in the following conferences:
  - International AIDS Society (IAS) Conference 2019 and 2020
  - Conference on Retroviruses and Opportunistic Infections (CROI) 2019 and 2020
  - International Workshop on HIV Pediatrics (IWoHP) 2018 and 2019
  - International Conference on AIDS and STIs in Africa (ICASA) 2019
- In the update run in March 2021, two additional conferences were searched:
  - CROI 2021
  - International Workshop on HIV Pediatrics 2020

Reference lists of systematic reviews identified via the electronic database searches were screened. Systematic reviews were only screened if they assessed one or more of the treatments of interest in a paediatric or adolescent population

**A2. Full search strategies**

Electronic databases search strategies

Original search strategies run in August 2020 and update searches were run in March 2021. Search strategies for Medline, Embase and the Cochrane Library (CENTRAL) are presented below. The review was developed to assess five drugs: dolutegravir, raltegravir, darunavir, lopinavir (solid formulations) and tenofovir alafenamide. The article presents the results pertaining to dolutegravir and raltegravir.

Medline search strategy run

Search strategy – Ovid MEDLINE(R) and Epub Ahead of Print, In-Process & Other Non-Indexed Citations and Daily

| # | Searches |
| --- | --- |
| 1 | exp HIV/ |
| 2 | exp HIV Infections/ |
| 3 | (HIV or HIV1 or HIV-1 or HIV2 or HIV-2 or human immun?deficiency virus* or human immun? deficiency virus*).ti,ab,kf. |
| 4 | (AIDS or acquired immun? deficiency syndrome* or acquired immun?deficiency syndrome*).ti,ab,kf. |
| 5 | or/1-4 |
| 6 | exp Integrase Inhibitors/ |
| 7 | (integrase adj3 inhibitor*).ti,ab,kf. |
| 8 | exp HIV Protease Inhibitors/ |
| 9 | ((Protease or proteinase) adj3 inhibitor*).ti,ab,kf. |
| 10 | (dolutegravir or Tivicay or a613043 or a 613043 or GSK1349572 or GSK 1349572 or DTG).ti,ab,kf. |
| 11 | ((Tenofovir and alafenamide) or TAF or GS 7340 or GS7340 or vemlidy).ti,ab,kf. |
| 12 | (darunavir or Prezista or TMC114 or TMC 114 or a607042 or a 607042 or uic 94017 or uic94017 or DRV).ti,ab,kf. |
| 13 | exp Darunavir/ |
| 14 | (raltegravir or Isentress or MK0518 or "MK 0518" or a608004 or a 608004 or RAL or l 900612 or l900612).ti,ab,kf. |
| 15 | exp Raltegravir Potassium/ |
| 16 | (lopinavir or ABT378 or ABT 378 or a602015 or a 602015 or LPV or a 157378 or a157378).ti,ab,kf. |
| 17 | exp Lopinavir/ |
| 18 | or/6-17 |
| 19 | exp adolescent/ or exp child/ or exp infant/ |
| 20 | (infant disease* or childhood disease*).ti,ab,kf. |
| 21 | (adolescen* or babies or baby or boy? or boyfriend or boyhood or girlfriend or girlhood or child* or girl? or infan* or juvenil* or kid? or minors or minors* or neonat* or neo-nat* or newborn* or new-born* or paediatric* or peadiatric* or pediatric* or perinat* or preschool* or puber* or pubescen* or school* or teen* or toddler? or underage? or under-age? or youth* or young).ti,ab,kf. |
| 22 | (pediatric* or paediatric* or infan* or child* or adolescen* or young).jn,jw. |
| 23 | or/19-22 |
| 24 | 5 and 18 and 23 |
| 25 | (healthy adj3 (volunteer* or subject*)).ti,kf. |
| 26 | (Case Reports or Clinical Conference or "Clinical Trial, Veterinary" or Consensus Development Conference or Editorial or Guideline or Interview or Letter or Meeting Abstract or News or Newspaper Article or "Randomized Controlled Trial, Veterinary").pt. |
| 27 | (Case report* or case stud*).ti,kf. |
| 28 | (cost minimi* or cost-utilit* or health utility* or economic evaluation* or economic review* or cost outcome or cost analys?s or economic analys?s or budget* impact analys?s).ti,kf. |
| 29 | (Review not Systematic review).pt. |
| 30 | Animals/ not Humans/ |
| 31 | or/25-30 |
| 32 | 24 not 31 |
| 33 | limit 32 to english language |
| 34 | limit 32 to french |
| 35 | 33 or 34 |
| 36 | limit 35 to yr="2009 -Current" |

Embase search strategy

Search strategy – Embase

| # | Searches |
| --- | --- |
| 1 | exp Human immunodeficiency virus/ |
| 2 | exp Human immunodeficiency virus infection/ |
| 3 | exp Human immunodeficiency virus infected patient/ |
| 4 | (HIV or HIV1 or HIV-1 or HIV2 or HIV-2 or human immun?deficiency virus* or human immun? deficiency virus*).ti,ab,kw. |
| 5 | (AIDS or acquired immun? deficiency syndrome* or acquired immun?deficiency syndrome*).ti,ab,kw. |
| 6 | or/1-5 |
| 7 | exp integrase inhibitor/ |
| 8 | (integrase adj3 inhibitor*).ti,ab,kw. |
| 9 | exp Human immunodeficiency virus proteinase inhibitor/ |
| 10 | ((Protease or proteinase) adj3 inhibitor*).ti,ab,kw. |
| 11 | exp dolutegravir/ |
| 12 | (dolutegravir or Tivicay or a613043 or a 613043 or GSK1349572 or GSK 1349572 or DTG).ti,ab,kw. |
| 13 | exp tenofovir alafenamide/ |
| 14 | ((Tenofovir and alafenamide) or TAF or GS 7340 or GS7340 or vemlidy).ti,ab,kw. |
| 15 | exp darunavir/ |
| 16 | (darunavir or Prezista or TMC114 or TMC 114 or a607042 or a 607042 or uic 94017 or uic94017 or DRV).ti,ab,kw. |
| 17 | exp raltegravir/ |
| 18 | (raltegravir or Isentress or MK0518 or "MK 0518" or a608004 or a 608004 or RAL or l 900612 or l900612).ti,ab,kw. |
| 19 | exp lopinavir/ |
| 20 | (lopinavir or ABT378 or ABT 378 or a602015 or a 602015 or LPV or a 157378 or a157378).ti,ab,kw. |
| 21 | or/7-20 |
| 22 | exp adolescence/ or exp adolescent/ or exp child/ or exp childhood disease/ or exp infant disease/ |
| 23 | (adolescen* or babies or baby or boy? or boyfriend or boyhood or girlfriend or girlhood or child* or girl? or infan* or juvenil* or juvenile* or kid? or minors or minors* or neonat* or neo-nat* or neo-nat* or newborn* or new-born* or paediatric* or peadiatric* or pediatric* or perinat* or preschool* or puber* or pubescen* or school or school child* or school* or schoolchild* or schoolchild* or teen* or toddler? or underage? or under-age? or youth* or young).ti,ab,kw. |
| 24 | (pediatric* or paediatric* or infan* or child* or adolescen* or young).jn,jw. |
| 25 | or/22-24 |
| 26 | 6 and 21 and 25 |
| 27 | animal/ not human/ |
| 28 | (healthy adj3 (volunteer* or subject*)).ti,kw. |
| 29 | (Conference Abstract or Conference Review or Editorial or Letter or Note).pt. |
| 30 | (Case report* or case stud*).ti,kw. |
| 31 | (cost minimi* or cost-utilit* or health utility* or economic evaluation* or economic review* or cost outcome or cost analys?s or economic analys?s or budget* impact analys?s).ti,kw. |
| 32 | review.pt. not (systematic review* or meta analysis or metaanalysis).ti,kw. |
| 33 | or/27-32 |
| 34 | 26 not 33 |
| 35 | limit 34 to english language |
| 36 | limit 34 to french |
| 37 | 35 or 36 |
| 38 | limit 37 to yr="2009 -Current" |

Cochrane Library (CENTRAL) search strategy

Search strategy – Cochrane library

| ID | Search |
| --- | --- |
| #1 | MeSH descriptor: [HIV] explode all trees |
| #2 | MeSH descriptor: [HIV Infections] explode all trees |
| #3 | ((HIV or HIV1 or HIV-1 or HIV2 or HIV-2 or human immun?deficiency virus* or human immun? deficiency virus*)):ti,ab,kw |
| #4 | (AIDS or acquired immun? deficiency syndrome* or acquired immun?deficiency syndrome*):ti,ab,kw |
| #5 | OR #1-#4 |
| #6 | MeSH descriptor: [Integrase Inhibitors] explode all trees |
| #7 | (integrase NEAR/3 inhibitor*):ti,ab,kw |
| #8 | MeSH descriptor: [HIV Protease Inhibitors] explode all trees |
| #9 | (((Protease or proteinase) NEAR/3 inhibitor*)):ti,ab,kw |
| #10 | (dolutegravir or Tivicay or a613043 or a 613043 or GSK1349572 or GSK 1349572 or DTG):ti,ab,kw |
| #11 | ((Tenofovir and alafenamide) or TAF or GS 7340 or GS7340 or vemlidy):ti,ab,kw |
| #12 | (darunavir or Prezista or TMC114 or TMC 114 or a607042 or a 607042 or uic 94017 or uic94017 or DRV):ti,ab,kw |
| #13 | MeSH descriptor: [Darunavir] explode all trees |
| #14 | (raltegravir or Isentress or MK0518 or "MK 0518" or a608004 or a 608004 or RAL or l 900612 or l900612):ti,ab,kw |
| #15 | MeSH descriptor: [Raltegravir Potassium] explode all trees |
| #16 | (lopinavir or ABT378 or ABT 378 or a602015 or a 602015 or LPV or a 157378 or a157378):ti,ab,kw |
| #17 | MeSH descriptor: [Lopinavir] explode all trees |
| #18 | OR #6-#17 |
| #19 | MeSH descriptor: [Adolescent] explode all trees |
| #20 | MeSH descriptor: [Child] explode all trees |
| #21 | MeSH descriptor: [Infant] explode all trees |
| #22 | (infant disease* or childhood disease*):ti,ab,kw |
| #23 | (adolescen* or babies or baby or boy? or boyfriend or boyhood or girlfriend or girlhood or child* or girl? or infan* or juvenil* or kid? or minors or minors* or neonat* or neo-nat* or newborn* or new-born* or paediatric* or peadiatric* or pediatric* or perinat* or preschool* or puber* or pubescen* or school* or teen* or toddler? or underage? or under-age? or youth* or young):ti,ab,kw |
| #24 | OR #19-#23 |
| #25 | #5 AND #18 AND #24 |
| #26 | #25 with Publication Year from 2009 to 2020, in Trials |
| #27 | #25 in Cochrane Reviews |
| #28 | #26 or #27 |

Grey literature search strategies

Clinicaltrial.gov search strategy

In the advanced search settings (<https://clinicaltrials.gov/ct2/search/advanced>), the following terms were used:

**Condition or disease:** HIV

**Study type:** All studies

**Study results:** All studies

**Age group:** Child (birth-17)

**Intervention/treatment:** Dolutegravir OR DTG OR Tenofovir alafenamide OR TAF OR Darunavir OR DRV OR Raltegravir OR RAL OR Lopinavir OR LPV

**Last Update Posted:** From 01/01/2018; To 04/08/2021

All other fields were left at the default settings. Test search retrieved 67 studies (run 08 April 2021).

67 Studies found for: HIV | Dolutegravir OR DTG OR Tenofovir alafenamide OR TAF OR Darunavir OR DRV OR Raltegravir OR RAL OR Lopinavir OR LPV | Child | Last update posted from 01/01/2018 to 04/08/2021

Applied Filters: Child (birth–17)

WHO International Clinical Trials Registry search strategy

Website being updated to cope with increase traffic due to the COVID-19 pandemic. In the advanced search settings in the search platform under development (<https://ictrptest.azurewebsites.net/AdvSearch.aspx>), the following terms were used:

**In the Condition:** HIV

**In the Intervention:** Dolutegravir OR DTG OR Tenofovir alafenamide OR TAF OR Darunavir OR DRV OR Raltegravir OR RAL OR Lopinavir OR LPV

Selected tick box for “Search for clinical trials in children” and “Recruitment status” “ALL”. All other settings used were the default. The search results were exported into Excel and only trials which were “Last refreshed on” 2018 to Present were retained. Trials with NCT registration (i.e. from clinicaltrials.gov) were removed as the clinicaltrials.gov website will be more up to date.

182 records for 129 trials found.

EudraCT search strategy

In the advanced settings (<https://www.clinicaltrialsregister.eu/ctr-search/search>), the following terms were used:

**Search terms:** HIV AND (dolutegravir OR DTG OR Tenofovir alafenamide OR TAF OR DRV OR darunavir OR Raltegravir OR RAL OR Lopinavir OR LPV)

**Age range:** Adolescent and children and infant and toddler and newborn and preterm new born infants and under 18

All other fields were left blank. Search retrieved 47 trials with a EudraCT protocol.

47 result(s) found for: HIV AND (dolutegravir OR DTG OR Tenofovir alafenamide OR TAF OR DRV OR darunavir OR Raltegravir OR RAL OR Lopinavir OR LPV).

**A3. Data extraction**

Table. Complete list of efficacy/effectiveness and safety outcomes extracted in the systematic review, and where the data are reported in this review

| **Characteristic** | **Variables extracted** | **Were data identified in one or more included studies for this variable?** | **Location if reported in this systematic review** |  |
| --- | --- | --- | --- | --- |
| **Efficacy and effectiveness** | | | | |
| CD4 cell count – change from baseline | Change, 95% confidence interval, adjustment | Yes | Main body and Supporting file 2 |  |
| CD4 cell count and CD4% – absolute values | Mean, median, standard deviation, range, interquartile range | Yes | Inconsistently reported. Change in CD4 percent from baseline reported instead for children <6 years and change in CD4 count from baseline reported for children and adolescents ≥6 years |  |
| CD4 cell count categories | Categorical variable with n and % | Yes |  |  |
| HIV viral load thresholds | Categorical variable with n and % | Yes | Main body and Supporting file 2 |  |
| HIV viral load – absolute values | Mean, median, standard deviation, range, interquartile range | Yes | Inconsistently reported, viral load threshold data reported instead |  |
| HIV viral load – change from baseline | Change, 95% confidence interval, adjustment | Yes |  |  |
| **Safety** | | | | |
| All-cause mortality | n, % | Yes | Main body |  |
| Discontinuation | n, % | Yes | Main body |  |
| On treatment at end of follow-up | n, % | Yes | Discontinuation data reported instead |  |
| Time to discontinuation (months) | Median, interquartile range | No | - |  |
| Time to discontinuation | Categorical variable with n and % | No | - |  |
| Hospitalisation | n, % | Yes | Only reported in ODYSSEY tuberculosis sub-study: nine hospitalisations related to serious adverse events among 31 children and adolescents receiving dolutegravir and followed-up for 31 weeks |  |
| Grade 3/4 clinical and laboratory events | n, % | Yes | Main body |  |
| Grade 3/4 adverse drug reactions | n, % | Yes | Main body |  |
| AIDS-related events | n, % | Yes | Reported in five studies, one event reported. |  |
| Fractures | Location, n, % | No | - |  |
| Bone mineral density | Mean, median, standard deviation, range, interquartile range, change from baseline, 95% confidence interval, adjustment | No | - |  |
| Estimated glomerular filtration rate | Mean, median, standard deviation, range, interquartile range, change from baseline, 95% confidence interval, adjustment | No | - |  |
| Creatinine clearance | Mean, median, standard deviation, range, interquartile range, change from baseline, 95% confidence interval, adjustment | Yes | Not reported here as only one study was identified for dolutegravir and raltegravir. |  |
| Grade 3/4 psychiatric effects | n, % | Yes | Main body |  |
| Neurological development | Yes/no. If yes, list measurement used | No | - |  |
| Growth – height for age Z-score (HAZ) | Mean, median, standard deviation, range, interquartile range, point estimate, 95% confidence interval, adjustment | Yes | Main body |  |
| Growth – HAZ categorical variables | Categorical variable, n, % | No | - |  |
| Weight for age Z-score (WAZ) | Mean, median, standard deviation, range, interquartile range, point estimate, 95% confidence interval, adjustment | Yes | Main body |  |
| WAZ categorical variables (stunting, underweight, wasting, and overweight) | Variable definition, n, % | No | - |  |
| High blood pressure | Definition, n, % | No | - |  |
| Hyperglycaemia | Definition, n, % | No | - |  |
| Diabetes | Definition, n, % | No | - |  |
| Elevated bilirubin | Mean, median, standard deviation, range, interquartile range, point estimate, 95% confidence interval, adjustment | No | - |  |
| Lipids (total cholesterol, low-density lipoprotein, high-density lipoprotein, triglycerides) | Mean, median, standard deviation, range, interquartile range, point estimate, 95% confidence interval, adjustment | Yes | Main body |  |
| Hypersensitivity reaction | Definition, n, % | No | - |  |

**References**

1. World Health Organization. Consolidated guidelines on the use of antiretroviral drugs for treating and preventing HIV infection: recommendations for a public health approach – 2nd ed. France: World Health Organization; 2016. Available from: <https://apps.who.int/iris/handle/10665/208825> (Accessed 15/01/2022).

2. US National Institutes of Health. Guidelines for the Use of Antiretroviral Agents in Pediatric HIV Infection. 2020. Available from: <https://clinicalinfo.hiv.gov/en/guidelines/pediatric-arv/whats-new-guidelines> (Accessed 21/03/2020).

3. Bamford A, Turkova A, Lyall H, Foster C, Klein N, Bastiaans D, et al. Paediatric European Network for Treatment of AIDS (PENTA) guidelines for treatment of paediatric HIV-1 infection 2015: optimizing health in preparation for adult life. HIV Med. 2018;19(1):e1-e42.

4. Foster C, Bamford A, Turkova A, Welch S, Klein N. Paediatric European Network for Treatment of AIDS Treatment Guideline 2016 update: antiretroviral therapy recommended for all children living with HIV. HIV Med. 2017;18(2):133-4.

5. Paediatric European Network for Treatment of AIDS. PENTA HIV First and second line antiretroviral treatment guidelines 2019. 2019. Available from: <https://penta-id.org/news/guidelines/penta-hiv-1st-and-2nd-line-antiretroviral-treatment-guidelines-2019/> (Accessed 15/11/2021).
