# Supplementary material for: Effectiveness and safety of dolutegravir and raltegravir for treating children and adolescents living with HIV: a systematic review
Source: J Int AIDS Soc. 2022 Nov 14;25(11):e25970. doi: 10.1002/jia2.25970 (PMC9663860; doi:10.1002/jia2.25970)
Supplement: Supplementary file 2 — File S2. Supplementary tables. Table S1. Study characteristics of clinical trials on dolutegravir or raltegravir in infants, children and/or adolescents identified through searching of registries, March 2021, for which no publications were identified at the time of the searches. Table S2. Viral load data used to create the scatterplots presented in the paper (Figure 3). Table S3. Treatment failure data from ODYSSEY. Table S4. CD4 cell count or percent at baseline in studies in which data on change in CD4 measurement from baseline were also reported. Table S5. Change in mean or median CD4 cell count or CD4 percent from baseline. Table S6. Details of population sub‐groups presented in Figure 4 (age‐bands, weight groups and dose/formulations). [file JIA2-25-e25970-s002.docx]

**Supporting file 2. Supplementary tables**

**Table S1.** Study characteristics of clinical trials on dolutegravir or raltegravir in infants, children and/or adolescents identified through searching of registries, March 2021, for which no publications were identified at the time of the searches

| **Trial number (study name and design)** | **Title** | **Treatment** | **Completion date** | **URL** |
| --- | --- | --- | --- | --- |
| **DOLUTEGRAVIR** |  |  |  |  |
| ISRCTN85058577 (Breather Plus, RCT) | Breather Plus: A randomised open-label 2-arm, 96-week trial evaluating the efficacy, safety and acceptability of short cycle (five days on, two days off) dolutegravir/tenofovir-based triple antiretroviral therapy (ART) compared to daily dolutegravir/tenofovir-based triple ART in virologically suppressed HIV-infected adolescents aged 12 to 19 years of age in sub-Saharan Africa | Control group: Continuous dolutegravir, with a tenofovir and lamivudine/emtricitabine backbone; Experimental group: Short-cycle dolutegravir, with a tenofovir and lamivudine/emtricitabine backbone (5 consecutive days on ART, 2 consecutive days off every week. | 31/12/2024 | https://trialsearch.who.int/Trial2.aspx?TrialID=ISRCTN85058577 |
| TCTR20180817004 (Single-arm) | An open label, single arm study of the safety and efficacy of DTG/3TC in therapy-naive HIV-1 infected adolescents. | Dolutegravir/lamivudine (DTG/3TC) as a fixed dose combination (FDC), 50 mg/300 mg fixed dose combination (FDC) Tablet, Once daily | 31/10/2022 | www.thaiclinicaltrials.org/show/TCTR20180817004 |
| TCTR20201025001 (Single-arm) | Pharmacokinetic, safety, and efficacy of dolutegravir dispersible tablet in young children living with HIV. | DTG dispersible tablet as their weight band dosing 20 mg for weight 6-<10, 10-<14 kg, and 25 mg for weight 14-<20kg | 31/07/2021 | www.thaiclinicaltrials.org/show/TCTR20201025001 |
| PACTR202008554705202 (Single-arm) | An open-label, sequential non-randomised pharmacokinetics study of dolutegravir (DTG) plasma exposure when given as twice or once daily DTG in the presence of rifampicin in children with Human Immune Deficiency Virus (HIV) and tuberculosis (TB) between 20-35kgs in South Africa. (Stage 1 using twice daily in accordance with the standard of care dosing). | DTG BID 50 mg | Not reported | https://pactr.samrc.ac.za/TrialDisplay.aspx?TrialID=12232 |
| ISRCTN17157458/NCT04337450 (RCT) | A randomised non-inferiority trial with nested PK to assess DTG/3TC fixed-dose formulations for the maintenance of virological suppression in children with HIV infection aged 2 to <15 years | Standard-of-care (SOC): 2 nucleo(s/t)ide reverse transcriptase inhibitor (NRTI) and a third (anchor) drug (either an integrase strand transfer inhibitor (INSTI), a protease inhibitor (PI) or a non-nucleoside reverse transcriptase inhibitor (NNRTI). Experimental: Once-daily DTG/3TC fixed-dose combination dispersible or film-coated tablets dosed using WHO weight bands criteria. | 30/04/2025 | https://trialsearch.who.int/Trial2.aspx?TrialID=ISRCTN17157458 |
| NCT02383108 (SMILE (PENTA 17)\|ANRS1 52, RCT) | A Two-arm, Phase 2/3 Multicentre, Open-label, Randomised Study Evaluating Safety and Antiviral Effect of Current Standard Antiretroviral Therapy Compared to Once Daily Integrase Inhibitor Administered With Darunavir/Ritonavir (DRV/r) in HIV-1 Infected, Virologically Suppressed Paediatric Participants. | Drug: Once daily integrase inhibitor (INSTI) + darunavir/ritonavir (DRV/r) (NRTI-sparing regimen). Standard of care: continuing triple antiretroviral therapy including 2 NRTIs + boosted PI/NNRTI. | 01/10/2020 | https://ClinicalTrials.gov/show/NCT02383108 |
| NCT03682848 (Single-arm) | An Open-label, Single Arm Study to Evaluate the Week 48 Efficacy and Safety of a Two-drug Regimen of Dolutegravir/Lamivudine (DTG/3TC) as a Fixed Dose Combination (FDC), in Antiretroviral Therapy (ART)-Naive HIV-1-infected Adolescents, ≥12 to <18 Years of Age Who Weigh at Least 25 kg | DTG + 3TC FDC will be available as 50/300 milligrams tablet to be given orally once daily. | 29/09/2026 | https://ClinicalTrials.gov/show/NCT03682848 |
| NCT03760458 (IMPAACT 2019, Single-arm) | The Pharmacokinetics, Safety, and Tolerability of Abacavir/Dolutegravir/Lamivudine Dispersible and Immediate Release Tablets in HIV-1-Infected Children Less Than 12 Years of Age | Drug: Abacavir (ABC)/Dolutegravir (DTG)/Lamivudine (3TC) Dispersible Tablets\|Drug: Abacavir (ABC)/Dolutegravir (DTG)/Lamivudine (3TC) Immediate Release Tablets (Immediate release) | 17/06/2024 | https://ClinicalTrials.gov/show/NCT03760458 |
| NCT04337450 (D3 (Penta21), RCT) | A Randomised Non-inferiority Trial With Nested PK to Assess DTG/3TC Fixed Dose Formulations for the Maintenance of Virological Suppression in Children With HIV Infection Aged 2 to <15 Years Old | Dolutegravir (DTG) and lamivudine (3TC); or standard of care: 2 nucleos(t)ide reverse transcriptase inhibitor (NRTI) and a third (anchor) drug (either an integrase strand transfer inhibitor (INSTI), a protease inhibitor (PI) or a non- nucleoside reverse transcriptase inhibitor (NNRTI); | 01/04/2025 | https://ClinicalTrials.gov/show/NCT04337450 |
| NCT03016533 (IMPAACT P1093 and P2019, Single-arm) | Open-label Access to Dolutegravir for HIV-1 Infected Children and Adolescents Completing IMPAACT Studies P1093 and P2019 | Drug: Dolutegravir film-coated tablets\|Drug: Dolutegravir film-coated dispersible tablets\|Drug: ABC/DTG/3TC immediate release tablets\|Drug: ABC/DTG/3TC film-coated dispersible tablets | 30/09/2024 | https://ClinicalTrials.gov/show/NCT03016533 |
| NCT04746547 (CAPRISA258 (CAP258), Single-arm) | An Open-label, Sequential Non-randomised Pharmacokinetics Study of DTG Plasma Exposure When Given as Twice or Once Daily DTG in the Presence of Rifampicin in Children With HIV and TB Between 20-35kgs in SA | Twice daily dolutegravir with rifampicin containing TB treatment | 31/08/2022 | https://ClinicalTrials.gov/show/NCT04746547 |
| NCT03602690 (ANRS 12374 (3DICAM), Single-arm) | Dolutegravir, Darunavir/Ritonavir and Optimized NRTI Recycling as a Third-line Antiretroviral Regimen in Cambodia | Dolutegravir 50mg once daily Darunavir/ritonavir 600/100 twice daily Optimized NRTI recycling with lamivudine and one other NRTI (zidovudine or tenofovir or abacavir) | 11/04/2022 | https://ClinicalTrials.gov/show/NCT03602690 |
| NCT04050449 (Hakim Study, ACTG A5381, Observational) | Observational Cohort to Assess Therapeutic Efficacy and Emergence of HIV Drug Resistance Following Initiation of Tenofovir-Lamivudine-Dolutegravir (TLD) for First- or Second-Line ART or With Rifampin-Containing TB Treatment: The Hakim Study | Tenofovir disoproxil fumarate/lamivudine/dolutegravir (TLD) | 31/05/2024 | https://ClinicalTrials.gov/show/NCT04050449 |
| **RALTEGRAVIR** |  |  |  |  |
| NCT01717287 (0518-248, Single-arm trial) | A Study of the Safety, Tolerability, and Antiretroviral Activity of Raltegravir (MK-0518) in Combination With Other Antiretroviral Therapies in Russian Children and Adolescents Infected With Human Immunodeficiency Virus (HIV-1) (MK-0518-248) | Drug: Raltegravir Film-coated Tablet\|Drug: Raltegravir Chewable Tablet\|Drug: Other Anti-Retroviral Therapy | 11/12/2013 | [https://ClinicalTrials.gov/show/NCT01717287](https://clinicaltrials.gov/show/NCT01717287) |
| NCT02140255 (IMPAACT P1115, Single-arm trial) | Very Early Intensive Treatment of HIV-Infected Infants to Achieve HIV Remission: A Phase I/II Proof of Concept Study | Drug: Nucleoside Reverse Transcriptase Inhibitors (NRTIs)\|Drug: Nevirapine (NVP)\|Drug: Lopinavir/Ritonavir (LPV/r)\|Drug: Raltegravir (RAL)\|Drug: VRC01 | 31/12/2031 | [https://ClinicalTrials.gov/show/NCT02140255](https://clinicaltrials.gov/show/NCT02140255) |
| NCT01751568 (IMPAACT P1101, Single-arm trial) | Phase I/II Dose-Finding, Safety, Tolerance, and Pharmacokinetics Study of a Raltegravir-Containing Antiretroviral Therapy (ART) Regimen in HIV-Infected and TB Co-Infected Infants and Children | Chewable raltegravir tablets, initially dosed at 12 mg/kg (up to a maximum of 800 mg) orally twice daily. | 27/11/2019 | [https://ClinicalTrials.gov/show/NCT01751568](https://clinicaltrials.gov/show/NCT01751568) |

**Table S2.** Viral load data used to create the scatterplots presented in the paper (Figure 3)

| **Study name** | **Age category** | **Treatment experience** | **Additional population details** | **N on treatment** | **Follow-up (weeks)** | **Additional details on follow-up** | **Lowest HIV viral load category reported (copies/mL)** | **N with data** | **n** | **%** |
| --- | --- | --- | --- | --- | --- | --- | --- | --- | --- | --- |
| **DOLUTEGRAVIR – SINGLE-ARM TRIAL** | | |  |  |  |  |  |  |  |  |
| IMPAACT P1093 | Infants | Mixed | Age 4 weeks to <6 months (Cohort V) | 17 | 24 | - | <50 | 17 | 7 | 41 |
| IMPAACT P1093 | Infants and children | Unclear | Age 6 months to <2 years (Cohort IV) | 9 | 24 | - | <50 | 9 | 6 | 67 |
| IMPAACT P1093 | Children | Unclear | Age 2 to <6 years (Cohort III) | 8 | 24 | - | <50 | 8 | 5 | 63 |
| IMPAACT P1093 | Children | Second- or subsequent-line | Age 6-12 years (Cohort IIA) | 23 | 48 | 21 (91.3%) completed the 48-week study visit | <50 | 23 | 17 | 74 |
| IMPAACT P1093 | Adolescents | Second- or subsequent-line | - | 23 | 24 | - | <50 | 23 | 16 | 67 |
|  |  |  |  |  | 48 | - | <50 | 23 | 14 | 61 |
|  |  |  |  |  | 96 | - | <50 | 23 | 7 | 30 |
|  |  |  |  |  | 144 | - | <50 | 23 | 8 | 35 |
| **DOLUTEGRAVIR – OBSERVATIONAL STUDIES** | | | |  |  |  |  |  |  |  |
| Briand 2017 | Adolescents | Second- or subsequent-line | - | 50 | 40 | Median 9 months (IQR 5–13) | <50 | 50 | 39 | 78 |
| ANRS EPF-CO10 | Children and adolescents | Unclear | On InSTI (n=65 DTG) | 69 | >26 | 96% (157/163) of all children had been on combination ART for at least 6 months | <50 | 69 | 58 | 84 |
| Frange 2019 | Children (5-11 years) | Mixed | - | 33 | 69 | Median 16 months (IQR 10-43) | <50 | 33 | 31 | 94 |
| Frange 2019 | Adolescents (12-17 years) | Mixed | - | 51 | 104 | Median 24 months (IQR 9-56) | <50 | 51 | 43 | 84 |
| Bacha 2020 | Children and adolescents | Mixed | - | 681 | Up to 43 | Started DTG from March 2019 and were followed until Dec 2019 | <1000 | 593 | 499 | 84 |
| Iyer 2021 | Children | Unclear | <10 years | - | 156 | Kaplan-Meier graph: patients 'at risk' after 156 weeks | <1000 | - | - | 94 |
| Iyer 2021 | Children and adolescents | Unclear | 10-14 years | - | 156 | Kaplan-Meier graph: patients 'at risk' after 156 weeks | <1000 | - | - | 94 |
| Iyer 2021 | Adolescents | Unclear | 15-17 years | - | 156 | Kaplan-Meier graph: patients 'at risk' after 156 weeks | <1000 | - | - | 94 |
| **DOLUTEGRAVIR AND RALTEGRAVIR** | | |  |  |  |  |  |  |  |  |
| Abo 2019 | Adolescents | First-line | On DTG | 2 | 62 | Median treatment exposure for all DTG patients | <50 | 2 | 2 | 100 |
| CHIPS | Children and adolescents | Mixed | On DTG | 272 | 26 | - | <50 | 178 | 157 | 88 |
|  |  |  |  |  | 52 | - | <50 | 124 | 112 | 90 |
| CHIPS | Children and adolescents | First-line | On DTG | 28 | 26 | - | <50 | 14 | 14 | 100 |
|  |  |  |  |  | 52 | - | <50 | 12 | 11 | 92 |
| CHIPS | Children and adolescents | Mixed | On RAL | 99 | 26 | - | <50 | 67 | 44 | 66 |
|  |  |  |  |  | 52 | - | <50 | 53 | 44 | 83 |
| CHIPS | Children and adolescents | First-line | On RAL | 14 | 26 | - | <50 | 5 | 3 | 60 |
|  |  |  |  |  | 52 | - | <50 | 4 | 4 | 100 |
| **RALTEGRAVIR – SINGLE-ARM TRIAL** | | |  |  |  |  |  |  |  |  |
| IMPAACT P1066 | Infants and children | Mixed | 4 weeks to <2 years old on RAL granules for suspension | 26 | 24 | - | <50 | 25 | 9 | 36 |
|  |  |  |  |  | 48 | - | <50 | 24 | 10 | 42 |
|  |  |  |  |  | 96 | - | <50 | 23 | 11 | 48 |
|  |  |  |  |  | 144 | - | <50 | 21 | 11 | 52 |
|  |  |  |  |  | 192 | - | <50 | 16 | 5 | 31 |
|  |  |  |  |  | 240 | - | <50 | 15 | 7 | 47 |
| IMPAACT P1066 | Children | Second- or subsequent-line | 2 to <12 years on RAL chewable tablet | 33 | 24 | - | <50 | 33 | 17 | 52 |
|  |  |  |  |  | 48 | - | <50 | 30 | 17 | 57 |
|  |  |  |  |  | 96 | - | <50 | 31 | 16 | 52 |
|  |  |  |  |  | 144 | - | <50 | 32 | 16 | 50 |
|  |  |  |  |  | 192 | - | <50 | 32 | 20 | 63 |
|  |  |  |  |  | 240 | - | <50 | 31 | 17 | 55 |
| IMPAACT P1066 | Children and adolescents | Second- or subsequent-line | 6 to <19 years old (≥25 kg) on RAL film-coated tablet | 63 | 24 | - | <50 | 62 | 34 | 55 |
|  |  |  |  |  | 48 | - | <50 | 60 | 34 | 57 |
|  |  |  |  |  | 96 | - | <50 | 52 | 25 | 48 |
|  |  |  |  |  | 144 | - | <50 | 50 | 23 | 46 |
|  |  |  |  |  | 192 | - | <50 | 46 | 19 | 41 |
|  |  |  |  |  | 240 | - | <50 | 43 | 13 | 30 |
| **RALTEGRAVIR – OBSERVATIONAL STUDIES** | | |  |  |  |  |  |  |  |  |
| CoRISPe | Children and adolescents | Second- or subsequent-line | - | 19 | 80.1 | IQR 49.4-96.4 weeks | <50 | 19 | 13 | 68 |
| IeDEA Global Consortium | Children and adolescents | Second- or subsequent-line | - | 62 | 26 | Median duration on RAL: 2 years (IQR 0.8-3) | <400 | 48 | 35 | 73 |
|  |  |  |  |  | 52 |  | <400 | 53 | 37 | 70 |
| Ferreira 2019 | Children | Second- or subsequent-line | Age 2-4 years | 44 | Up to 52 | VL performed after 30 days and before 365 days after switch to RAL | <50 | 44 | 20 | 46 |
| Ferreira 2019 | Children | Second- or subsequent-line | Age 5-8 years | 90 | Up to 52 | VL performed after 30 days and before 365 days after switch to RAL | <50 | 90 | 57 | 63 |
| Ferreira 2019 | Children | Second- or subsequent-line | Age 9-12 years | 87 | Up to 52 | VL performed after 30 days and before 365 days after switch to RAL | <50 | 87 | 57 | 66 |
| Rozenszajn 2020 | Children and adolescents | Second- or subsequent-line | - | 41 | >52 | Median time on RAL regimen was greater than 1 year for both viral subtypes | Viral suppression | 41 | 31 | 76 |
| **RALTEGRAVIR +/- DARUNAVIR – OBSERVATIONAL STUDIES** | | | |  |  |  |  |  |  |  |
| Huerta-Garcia 2016 | Children and adolescents | Second- or subsequent-line | On optimised regimen (n=13 RAL; n=13 DRV) | 16 | 48 | - | <50 | 16 | 11 | 69 |
| Thuret 2009 | Adolescents | Second- or subsequent-line | On DRV/r+RAL+ETV | 12 | 26 | - | <50 | 12 | 5 | 42 |
|  |  |  |  |  | 39 | - | <50 | 12 | 5 | 42 |

DRV/r, ritonavir-boosted darunavir; DTG, dolutegravir; ETV, etravirine; InSTI, integrase inhibitor; IQR, interquartile range; RAL, raltegravir; VL, viral load

**Table S3.** Treatment failure* data from ODYSSEY

| **Study name** | **Age category** | **Treatment experience** | **Treatment** | **Follow-up (weeks)** | **N** | **n** | **%** |
| --- | --- | --- | --- | --- | --- | --- | --- |
| ODYSSEY | Children and adolescents | First-line | Dolutegravir | 96 | 154 | 15 | 10 |
|  |  |  | Standard of care | 96 | 157 | 34 | 23 |
| ODYSSEY | Children and adolescents | Second-line | Dolutegravir | 96 | 196 | 32 | 17 |
|  |  |  | Standard of care | 96 | 200 | 41 | 21 |

***** Treatment failure was defined as confirmed viral load ≥400 copies/mL after week 36, lack of virological response by 24 weeks with ART switch, death or new/recurrent WHO 4/severe WHO 3 event by 96 weeks.

**Table S4.** CD4 cell count or percent at baseline in studies in which data on change in CD4 measurement from baseline were also reported. Where data were available, CD4 counts and percentages are presented for populations <6 years old to account for their changing physiology; only CD4 counts are presented for populations ≥6 years old.

| Study name | Population | N* | Measurement | CD4 cell count (cells/µL) or percent at baseline | | | | | | | |
| --- | --- | --- | --- | --- | --- | --- | --- | --- | --- | --- | --- |
|  |  |  |  | Mean | Median | SD | Range/IQR | | Lower | | Upper |
| **DOLUTEGRAVIR** | |  |  |  |  |  | |  | |  |  |
| ODYSSEY | Children and adolescents (44% treatment naïve) receiving DTG (n=350) or SOC (n=357) | 707 | CD4 count | - | 459 | - | IQR | | 228 | | 704 |
| IMPAACT P1093 | Infants and children (29.4% treatment naïve) | 51 | CD4 count | - | 1866 | - | IQR | | 1189 | | 2384 |
|  |  | 51 | CD4% | - | 24.2 | - | IQR | | 20.0 | | 31.0 |
| IMPAACT P1093 | Children (6 to <12 years) | 23 | CD4 count | - | 645 | - | IQR | | 466 | | 732 |
| IMPAACT P1093 | Adolescents | 23 | CD4 count | - | 466 | - | IQR | | 297 | | 771 |
| CHIPS | Adolescents (10% treatment naïve) | 212 | CD4 count | - | 665 | - | IQR | | 470 | | 966 |
| **RALTEGRAVIR** |  |  |  |  |  |  |  | |  | |  |
| IMPAACT P1066 | Infants and children (4 weeks to 2 years) | 26 | CD4 count | - | 1400 | - | - | | - | | - |
|  |  | 26 | CD4% | - | 18.6 | - | - | | - | | - |
| IMPAACT P1066 | Children (2 to <6 years) | 20 | CD4 count | 1114.6 | 1086 | 549.7 | Range | | 323 | | 2361 |
|  |  | 20 | CD4% | 28.2 | 28.7 | 8.3 | Range | | 12.9 | | 41.8 |
| IMPAACT P1066 | Children (6 to <12 years) | 13 | CD4 count | 577.9 | 529 | 269.8 | Range | | 16 | | 1000 |
| IMPAACT P1066 | Children (6 to <12 years [≥25 kg]) | 4 | CD4 count | 850.5 | 806.5 | 509.5 | Range | | 274 | | 1515 |
| IMPAACT P1066 | Adolescents | 59 | CD4 count | 397.5 | 396.5 | 229.8 | Range | | 0 | | 872 |
| CHIPS | Children and adolescents (14% naïve) | 82 | CD4 count | - | 442 | - | IQR | | 190 | | 862 |
| CoRISPe | Adolescents | 19 | CD4 count | - | 329 | - | IQR | | 175 | | 452 |

* Where available, we extracted the number of patients with data. If not reported, total number of patients were extracted.

DTG, dolutegravir; IQR, interquartile range; SD, standard deviation; SOC, standard of care

**Table S5.** Change in mean or median CD4 cell count or percent from baseline

| **Study name** | **Population** | | **Follow-up (weeks)** | **N*** | **Estimate type (unit)** | **Change from baseline** | **IQR or 95% CI** | **Lower** | **Upper** |
| --- | --- | --- | --- | --- | --- | --- | --- | --- | --- |
| **DOLUTEGRAVIR** |  | |  |  |  |  |  |  |  |
| ODYSSEY | Children and adolescents (44% treatment naïve) | DTG | 48 | 350 | Mean (cells/µL) | 219† | - | - | - |
|  |  | SOC | 48 | 357 | Mean (cells/µL) | 189† | - | - | - |
|  |  | DTG | 96 | 350 | Mean (cells/µL) | 266† | - | - | - |
|  |  | SOC | 96 | 357 | Mean (cells/µL) | 230† | - | - | - |
| CHIPS | Adolescents (first-line) | | 26 | 11 | Median (cells/µL) | 207 | IQR | 20 | 288 |
|  |  | | 52 | 7 |  | 180 | IQR | 122 | 309 |
| CHIPS | Children and adolescents suppressed at baseline | | 26 | 68 | Median (cells/µL) | -27 | IQR | -73 | 93 |
|  |  |  | 52 | 52 |  | 39 | IQR | -97 | 136 |
| CHIPS | Children and adolescents unsuppressed at baseline | | 26 | 32 | Median (cells/µL) | 54 | IQR | -12 | 178 |
|  |  |  | 52 | 14 |  | 57 | IQR | -94 | 332 |
| IMPAACT P1093 | Infants (4 weeks to 6 months) | | 24 | 17 | Median (cells/µL) | 352 | IQR | -189 | 926 |
|  |  | | 24 | 17 | Median (CD4%) | 5 | IQR | 3 | 9 |
| IMPAACT P1093 | Infants and children (6 months to <2 years) | | 24 | 9 | Median (cells/µL) | -221 | IQR | -962 | 150 |
|  |  | | 24 | 9 | Median (CD4%) | 3 | IQR | -10 | 7 |
| IMPAACT P1093 | Children (2 to <6 years) | | 24 | 8 | Median (cells/µL) | 76 | IQR | -173 | 458 |
|  |  | | 24 | 8 | Median (CD4%) | 5 | IQR | -2 | 9 |
| IMPAACT P1093 | Children (6 to <12 years) | | 48 | 23 | Median (cells/µL) | 387 | IQR | 49 | 575 |
| IMPAACT P1093 | Adolescents | | 48 | 23 | Median (cells/µL) | 84 | IQR | -81 | 238 |
| **RALTEGRAVIR** |  | |  |  |  |  |  |  |  |
| IMPAACT P1066 | Infants – unsuccessful prophylaxis for VT | | 48 | 12 | Mean (cells/µL) | 876 | 95% CI | 362.7 | 1389.3 |
|  |  | | 48 | 12 | Mean (CD4%) | 8.7 | 95% CI | 2.7 | 14.8 |
| CHIPS | Children and adolescents (first-line) | | 26 | 4 | Median (cells/µL) | 423‡ | IQR | 217 | 976 |
|  |  |  | 52 | 4 |  | 256 | IQR | -69 | 594 |
| REALITY | Children and adolescents on standard ART (first-line) | | 24 | 33 | Mean (cells/µL) | 152.2 | 95% CI | 111.1 | 193.3 |
|  |  |  | 48 | 33 |  | 251.8 | 95% CI | 165 | 338.1 |
| REALITY | Children and adolescents on RAL-intensified ART (first-line) | | 24 | 39 | Mean (cells/µL) | 179.5 | 95% CI | 140.1 | 217.1 |
|  |  |  | 48 | 39 |  | 323 | 95% CI | 255.3 | 390.2 |
| IMPAACT P1066 | Infants and children (4 weeks to 2 years; 54% experienced) | | 24 | 26 | Mean (CD4%) | 7.5 | 95% CI | 4.4 | 10.6 |
|  |  |  | 48 | 26 |  | 7.3 | 95% CI | 3.8 | 10.9 |
|  |  |  | 96 | 26 |  | 9 | 95% CI | 4.2 | 13.9 |
|  |  |  | 144 | 26 |  | 8.1 | 95% CI | 3.1 | 13 |
|  |  |  | 192 | 26 |  | 7.9 | 95% CI | 2.7 | 13 |
|  |  |  | 240 | 26 |  | 8.6 | 95% CI | 3.2 | 14 |
| IMPAACT P1066 | Infants and children (6 months to <2 years) | | 48 | 14 | Mean (CD4%) | 6.4 | 95% CI | 1.4 | 11.3 |
|  |  |  | 48 | 14 | Mean (cells/µL) | 278.8 | 95% CI | -185.6 | 743.2 |
|  |  | |  |  |  |  |  |  |  |
| IMPAACT P1066 | Children (2 to <6 years) | | 24 | 20 | Mean (cells/µL) | 147.2 | 95% CI | -2.7 | 297.1 |
|  |  | | 48 | 20 |  | 158.1 | 95% CI | 11.7 | 304.4 |
|  |  | | 24 | 20 | Mean (CD4%) | 5.3 | 95% CI | 2.9 | 7.7 |
|  |  | | 48 | 20 |  | 4.3 | 95% CI | 1 | 7.6 |
|  | Children (6 to <12 years) | | 24 | 13 | Mean (cells/µL) | 143.4 | 95% CI | -12.9 | 299.6 |
|  |  | | 48 | 13 |  | 76.8 | 95% CI | -85.3 | 238.9 |
|  | Children (2 to <12 years) | | 24 | 33 | Mean (CD4%) | 3.5 | 95% CI | 1.3 | 5.8 |
|  |  | | 48 | 33 |  | 3.3 | 95% CI | 0.8 | 5.8 |
|  |  | | 96 | 33 |  | 5 | 95% CI | 1.4 | 8.6 |
|  |  | | 144 | 33 |  | 5.3 | 95% CI | 1.5 | 9 |
|  |  | | 192 | 33 |  | 5.8 | 95% CI | 2.2 | 9.4 |
|  |  | | 240 | 33 |  | 5.2 | 95% CI | 1.8 | 8.7 |
| IMPAACT P1066 | Children (6 to <12 years [≥25 kg]) | | 24 | 4 | Mean (cells/µL) | -35.8 | 95% CI | -348.8 | 277.3 |
|  |  |  | 48 | 4 |  | 189.5 | 95% CI | -154.2 | 533.2 |
| IMPAACT P1066 | Children and adolescents (6 to <19 years [≥25 kg]) | | 24 | 63 | Mean (CD4%) | 3.9 | 95% CI | 2.7 | 5.1 |
|  |  |  | 48 | 63 |  | 5.3 | 95% CI | 4 | 6.6 |
|  |  |  | 96 | 63 |  | 5.7 | 95% CI | 4.2 | 7.2 |
|  |  |  | 144 | 63 |  | 4.6 | 95% CI | 2.8 | 6.5 |
|  |  |  | 192 | 63 |  | 4.1 | 95% CI | 2.1 | 6.1 |
|  |  |  | 240 | 63 |  | 2.8 | 95% CI | 0.4 | 5.2 |
| CHIPS | Children and adolescents suppressed at baseline | | 26 | 14 | Median (cells/µL) | -79 | IQR | -258 | 211 |
|  |  |  | 52 | 13 |  | -121 | IQR | -375 | 170 |
|  | Children and adolescents unsuppressed at baseline | | 26 | 35 | Median (cells/µL) | 126 | IQR | 1 | 200 |
|  |  |  | 52 | 26 |  | 237 | IQR | 60 | 351 |
| IMPAACT P1066 | Adolescents | | 24 | 59 | Mean (cells/µL) | 114.4 | 95% CI | 73.7 | 155.1 |
|  |  | | 48 | 59 |  | 168.2 | 95% CI | 117.5 | 218.9 |
| CoRISPe | Adolescents | | 80.1 | 19 | - | -§ | - | - | - |

* Where available, we extracted the number of patients with data. If not reported, total number of patients was extracted. † Data were estimated from a graph using the online software Webplot Digitizer. 95% confidence intervals were presented in the figure; however, the confidence intervals were unclear and were not extracted. ‡ Communication with author: The figure in the poster shows a median of 301 [133, 545] but the correct median is 423 [IQR 217, 976]; § 17 (89%) subjects experienced significant increases in their CD4 cell count, ranging from 70% to 90% of the baseline values; two subjects still presented severe immunosuppression at follow-up.

CI, confidence interval; DTG, dolutegravir; IQR, interquartile range; RAL, raltegravir, SD, standard deviation; SOC, standard of care; VT, vertical transmission.

**Table S6.** Details of population sub-groups presented in Figure 4 in the paper (age-bands, weight groups, dose/formulations).

| **Study name** | **Population** | **Sub-group details (weight-band, age group, and/or formulation/dose)** | **Treatment line** | **Follow-up duration (weeks)** | **N** |
| --- | --- | --- | --- | --- | --- |
| ODYSSEY PK sub- study* | Children | 20 kg to <25 kg, 25 mg tablet | Mixed | 24 | 17 |
|  | Children | 20 kg to <25 kg, 30 mg dispersible tablet | Mixed | 24 | 10 |
|  | Children | 20 kg to <25 kg, 50 mg tablet | Second-line | 24 | 9 |
|  | Children + adolescents | 25 kg to <30 kg, 25 mg tablet | Mixed | 19 | 17 |
|  | Children + adolescents | 25 kg to <30 kg, 50 mg tablet | Mixed | 24 | 17 |
|  | Children + adolescents | 30 kg to <40 kg, 35 mg tablet | Mixed | 13.1 | 11 |
|  | Children + adolescents | 30 kg to <40 kg, 50mg tablet | Mixed | 24 | 11 |
| IMPAACT P1093 | Infants + children | Age 4 weeks to 6 years | Mixed | 24 | 51 |
|  | Children | Age 6 to <12 years | Second-line | 48 | 23 |
|  | Adolescents | Age 12 to <18 years | Second-line | 44 | 23 |
|  | Adolescents | Age 12 to <18 years | Second-line | 144 | 23 |
| IMPAACT P1066 | Infants | Age 4 weeks to <6 months (unsuccessful prophylaxis for vertical transmission) | First-line | 48 | 12 |
|  | Infants + children | Age 6 months to <2 years | Second-line | 48 | 14 |
|  | Infants + children | Age 4 weeks to <2 years | Mixed | 240 | 12+14 |
|  | Children | Age 2 to <6 years | Second-line | 48 | 20 |
|  | Children | Age 6 to <12 years | Second-line | 48 | 13 |
|  | Children | Age 2 to <12 years | Second-line | 240 | 20+13 |
|  | Children | Age 6 to <12 years (≥25 kg) | Second-line | 48 | 4 |
|  | Adolescents | Age 12 to <19 years | Second-line | 48 | 59 |
|  | Children + adolescents | Age 6 to <19 years (≥25 kg) | Second-line | 240 | 4+59 |

*Within the sub-study, two subjects included in the 20 kg to <25 kg weight band (25 mg tablet) also contributed safety data on the 30 mg dispersible tablet (n=1) or 50 mg tablet (n=1); the 17 subjects in the 25 kg to <30 kg weight band contributed safety data on both the 25 mg and 50 mg tablets, while the 11 subjects in the 30 kg to <40 kg weight band contributed data on both the 35 mg and 50 mg tablets.
